# An Integrative Pharmacology-Based Strategy to Uncover the Mechanism of Xiong-Pi-Fang in Treating Coronary Heart Disease with Depression

**DOI:** 10.3389/fphar.2021.590602

**Published:** 2021-04-01

**Authors:** Lihong Zhang, Yu Zhang, Mingdan Zhu, Limin Pei, Fangjun Deng, JinHong Chen, Shaoqiang Zhang, Zidong Cong, Wuxun Du, Xuefeng Xiao

**Affiliations:** ^1^ Tianjin University of Traditional Chinese Medicine, Tianjin, China; ^2^ Second Affiliated Hospital, Tianjin University of Traditional Chinese Medicine, Tianjin, China

**Keywords:** integrative pharmacology, serum pharmacochemistry, network pharmacology analysis, Xiong-Pi-Fang, coronary heart disease with depression

## Abstract

**Objectives:** This study aimed to explore the mechanism of Xiong-Pi-Fang (XPF) in the treatment of coronary heart disease (CHD) with depression by an integrative strategy combining serum pharmacochemistry, network pharmacology analysis, and experimental validation.

**Methods:** An ultrahigh performance liquid chromatography-quadrupole-time-of-flight tandem mass spectrometry (UPLC-Q-TOF/MS) method was constructed to identify compounds in rat serum after oral administration of XPF, and a component-target network was established using Cytoscape, between the targets of XPF ingredients and CHD with depression. Furthermore, Gene Ontology and Kyoto Encyclopedia of Genes and Genomes pathway enrichment analyses were performed to deduce the mechanism of XPF in treating CHD with depression. Finally, in a chronic unpredictable mild stress (CUMS)-and isoproterenol (ISO)-induced rat model, TUNEL was used to detect the apoptosis index of the myocardium and hippocampus, ELISA and western blot were used to detect the predicted hub targets, namely AngII, 5-HT, cAMP, PKA, CREB, BDNF, Bcl-2, Bax, Cyt-c, and caspase-3.

**Results:** We identified 51 compounds in rat serum after oral administration of XPF, which mainly included phenolic acids, saponins, and flavonoids. Network pharmacology analysis revealed that XPF may regulate targets, such as *ACE2*, *HTR1A*, *HTR2A*, *AKT1*, *PKIA*, *CREB1*, *BDNF*, *BCL2*, *BAX*, *CASP3*, cAMP signaling pathway, and cell apoptosis process in the treatment of CHD with depression. ELISA analysis showed that XPF decreased Ang-II content in the circulation and central nervous system, inhibited 5-HT levels in peripheral circulation, and increased 5-HT content in the central nervous system and cAMP content in the myocardia and hippocampus. Meanwhile, western blot analysis indicated that XPF could upregulate the expression levels of PKA, CREB, and BDNF both in the myocardia and hippocampus. TUNEL staining indicated that the apoptosis index of myocardial and hippocampal cells increased in CUMS-and ISO-induced CHD in rats under depression, and XPF could increase the expression of Bcl-2, inhibit the expression of Bax, Cyt-c, and caspase-3, and rectify the injury of the hippocampus and myocardium, which exerted antidepressant and antimyocardial ischemia effects.

**Conclusion:** Our study proposed an integrated strategy, combining serum pharmacochemistry and network pharmacology to investigate the mechanisms of XPF in treating CHD with depression. The mechanism of XPF in treating CHD with depression may be related to the activation of the cAMP signaling pathway and the inhibition of the apoptosis.

## Introduction

Coronary heart disease (CHD) is a chronic and complex disease that poses a serious threat to human health (Mozaffarian et al., 2016; Blais et al., 2020). Traditional risk factors are mainly related to hypertension, hyperlipidemia, diabetes, etc (Li C et al., 2020). Moreover, depression is another independent risk factor for CHD, as it has been shown to diminish the quality of life of patients with CHD and also increase the incidence and mortality of major adverse cardiac events (Su et al., 2018; de Heer et al., 2020). Increasing evidence suggests that the incidence of CHD with depression is increasing, ranging from approximately 15–18%, moreover, approximately 31% of these patients develop major depressive disorder (MDD) (Carney and Freedland, 2017). However, there are no effective chemicals to improve the quality of life and survival rate of patients with comorbid CHD and depression (Lee et al., 2018; Magaard et al., 2018).

The ideal medication for CHD with depression would be integrated, improving myocardial blood supply and regulating nervous system function at the same time (Yeung et al., 2014; Chen M et al., 2018; Xue et al., 2019). Xiong-Pi-Fang (XPF), a classical traditional Chinese medicine (TCM) formula, consists of Radix Bupleuri (Bupleurum chinense DC. and Bupleurum scorzonerifolium Willd.) 15 g; Ligusticum wallichii (Conioselinum anthriscoides 'Chuanxiong' and Ligusticum striatum DC.) 12 g; Rhizoma Cyperi (Cyperus rotundus L.) 12 g; Sanders (Santalum album L.) 9 g; Fructus aurantii (Citrus × aurantium L. and Citrus trifoliata L.) 9 g; Dried tangerine peel (Citrus × aurantium L.) 15 g; Pinellia ternata (Pinellia ternata (Thunb.) Makino) 15 g; Herba Menthae (Mentha haplocalyx Briq.) 10 g; Perilla Stem (Perilla frutescens (L.) Britton) 15 g; Poria Cocos (Wolfiporia extensa (Peck) Ginns) 15 g; Rhizoma Atractylodis Macrocephalae (Atractylodes Macrocephala Koidz) 15 g; Radix Glycyrrhizae (Glycyrrhiza uralensis Fisch.) 9 g. It is widely used in clinical practices, showing satisfactory therapeutic effects in relieving angina pectoris, chest pain, and depression symptoms (Hou et al., 2017). However, it is difficult to fully understand the therapeutic mechanism of XPF for treating CHD with depression solely using traditional pharmacological methods. Hence, an integrated pharmacology-based strategy including computer high-throughput analytical techniques and biological experimental tools was established in this study, which provides a helpful method to reveal the potential bioactive ingredients and modern pharmacological mechanism of TCM (Park et al., 2018; Fang et al., 2019).

Integrative pharmacology, combined with conventional pharmacology, network pharmacology, bioinformatics, and other disciplines, is the systematic study of the overall interactions between drugs and humans at the molecular, cell, organ, and network levels (Hart and Xie, 2016; Zhang R. et al., 2018; Zhang R. et al., 2019). Network pharmacology, characterized by integration, systematization, and emphasis on drug interaction (Yuan et al., 2017), predicts the biological molecular mechanism of drug treatment of diseases from a holistic perspective by constructing an interaction network between active ingredients and disease targets. However, most TCM-related databases provide information on chemical ingredients extracted *in vitro* (Zhang W et al., 2019). Previous studies have shown that the drug ingredients absorbed into the blood circulation may be the main active constituents (Chen et al., 2014; Li et al., 2016). Therefore, it is critical to combine serum pharmacochemistry with a network pharmacology strategy to accurately establish the network.

**FIGURE 1:**
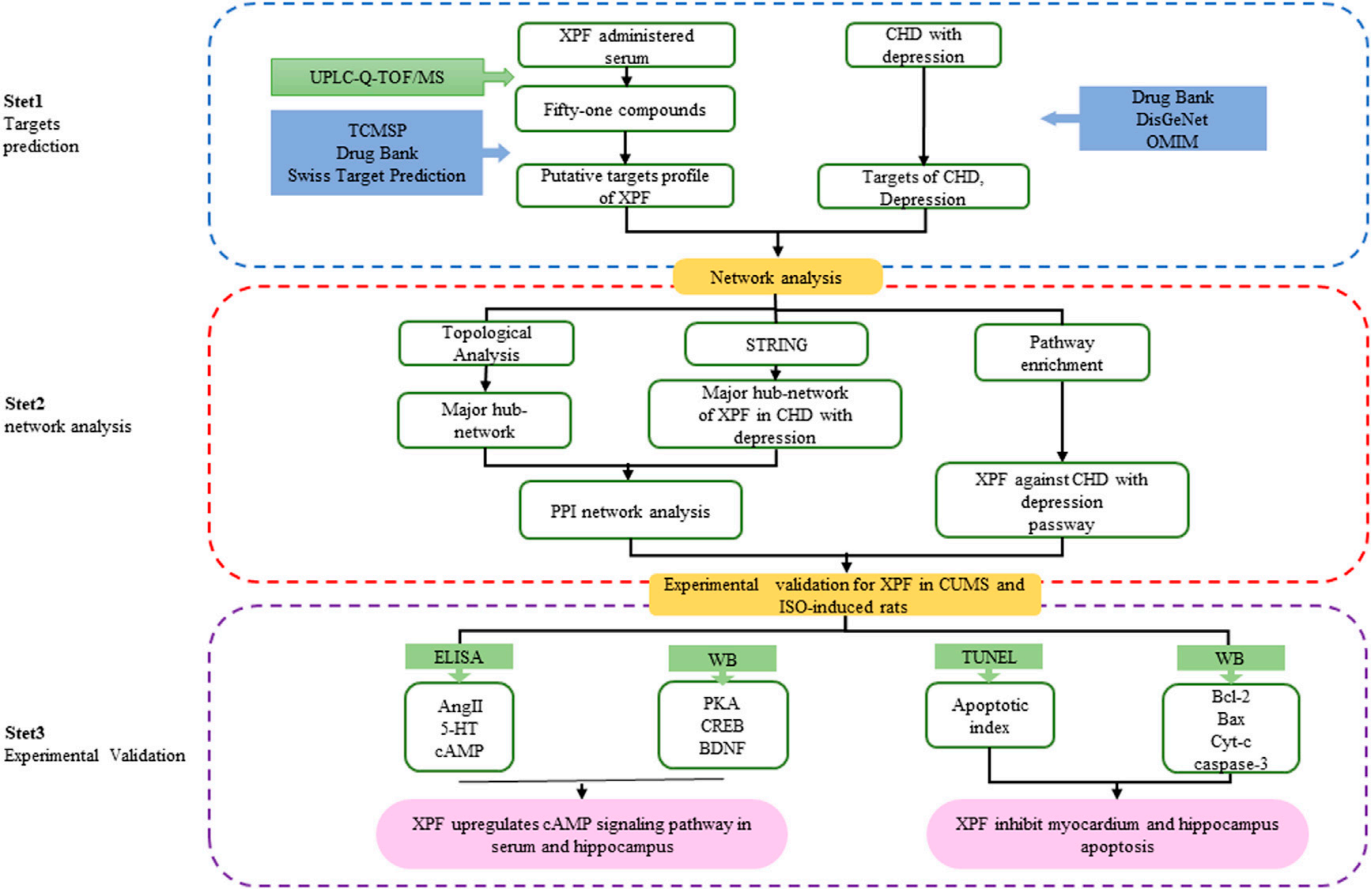
Flowchart of the study.

In this study, an integrated pharmacology strategy employing serum pharmacochemistry, network pharmacological analysis, and experimental validation was conducted to illustrate the therapeutic mechanism of XPF in treating CHD with depression (Figure 1). Briefly, An ultrahigh performance liquid chromatography-quadrupole-time-of-flight tandem mass spectrometry (UPLC-Q-TOF/MS) method was established to determine the main active components of XPF in rat serum. Furthermore, a serum pharmacochemistry-based network was constructed and the component–target network between CHD comorbid depression-relevant genes and the targets of active components in XPF was established. Finally, we validated the predicted molecular mechanisms obtained from the network analysis of XPF in treating CHD with depression in a chronic unpredictable mild stress (CUMS)-and isoproterenol (ISO)-induced CHD in a rat model of depression.

## Materials and Methods

### Reagents and Materials

Sertraline hydrochloride (Pfizer Inc., United States), metoprolol (Asilkan, United Kingdom), Isoproterenol (Sigma Aldrich Co., United States) were purchased from the Shanghai Yuanye Biotechnology Co. Ltd (Shanghai, China). UPLC-Q-TOF/MS grade acetonitrile and HPLC grade acetonitrile, methanol, formic acid, were provided by Fisher Scientific International Inc (Fair Lawn, NJ). The ELISA assay kits for Ang-Ⅱ (142191202), 5-HT (106191202), cAMP (202004) were purchased from Tianjin Zihan Biotechnology Co., Ltd. (Tianjin, China). TUNEL assay kit (7E310H9) was purchased from Vazyme Biotech Co., Ltd. (Nanjing, China), Rabbit antihuman monoclonal antibodies PKA (5842), CREB (6188), BDNF (3189), Bcl2 (26,593-1-12p), Bax (50,599–2-1 g), Cytochrome c (10993-1-Ap), and mouse antihuman monoclonal antibodies caspase-3 (66,470-E1g) and β-actin were provided by Proteintech Group, Inc. (Wuhan, China).

### Animals

Male Sprague-Dawley rats (weighing 200 ± 20 g) were purchased from the Experimental Animal Center of the Academy of Military Medical Sciences (Beijing, China), and kept in an environmentally controlled room (temperature 22 ± 2°C, humidity 50 ± 10%) with food and water available. This study was carried out in accordance with the principles of the Basel Declaration and the guidelines of the National Institutes of Health. All animal experiments were approved by the Ethics Committee of Tianjin University of Traditional Chinese Medicine (Tianjin, China).

### UPLC-Q-TOF/MS Method for Serum Pharmacochemistry Analysis

XPF was prepared by the pharmacy of the Second Affiliated Hospital of Tianjin University of Traditional Chinese Medicine (Tianjin, China). Twelve rats were randomly divided into a control group (*n* = 6, 0.9% saline IG), and According to the calculation method of equivalent dose coefficient of experimental animals in *Methodology of Pharmacological Experimen*t (Xu et al., 2018), the XPF group (*n* = 6, 32.7 g/kg XPF IG), equivalent to twice the clinical effective dose, for three days (Xu et al., 1997). All rats fasted for 12 h before the experiment and then the serum samples were collected at 120 min after oral administration, via the postorbital venous plexus. The serum samples were centrifuged at 12,000 rpm for 10 min at 4°C. Then, 300 μL of serum was added to three times the amount of acetonitrile, vortexed for 2 min, followed by ultrasonic extraction for 10 min, and centrifugation (12,000 rpm, 10 min). and The supernatant was collected and placed in a N_2_ blower to blow dry at 40°C, 50% acetonitrile (50 μL) was added to the residue, vortexed for 3 min, ultrasound for 10 min, centrifugation (12,000 rpm, 10 min), and the supernatant was collected for UPLC-Q-TOF/MS analysis.

UPLC-Q-TOF/MS analysis was analyzed on a Waters ACQUITYTM UPLC BEH C18 (100 mm × 2.1 mm, 1.7 μm) system, maintained at 30 °C. The flow rate was set at 0.45 ml/min, and the injection volume was 5 µL. The mobile phase was consisted of water (A) and methanol (B) both containing 0.1% (v/v) formic acid for astragaloside (0–5 min, 2% B; 5–8 min, 2%–20% B, 8–11 min, 20% B, 11–14 min, 20%–48% B, 14–20 min, 48%–70% B, 20–23 min, 70%–90% B, 23–26 min, 90%–100% B, 26–30 min, 100% B). The mass spectrometer analysis was performed with reaction monitoring both in positive and negative ion modes.

### Construction of the Compound-Target and Disease-Target Networks

All targets of active components of XPF determined by serum pharmacochemistry analysis were collected from TCM-related databases, including TCMSP (http://tcmspw.com/tcmsp.php) (Ru et al., 2014), DrugBank (https://www.drugbank.ca/) (Wishart et al., 2018), Swiss Target Prediction (http://www.swisstargetprediction.ch/) (Gfeller et al., 2014), and Similarity Ensemble Approach (http://sea.bkslab.org/) (Wang et al., 2016). Known therapeutic targets related to CHD and depression were obtained from the DrugBank database (Wishart et al., 2018), Online Mendelian Inheritance in Man (OMIM) (http://www.omim.org) (Amberger and Hamosh, 2017), DisGeNet database (http://www.disgenet.org/web/DisGeNET/menu/home) (Li Y et al., 2020), and Therapeutic Target database (TTD) (https://db.idrblab.org/ttd/) (Li et al., 2018). The common potential target of XPF in the treatment of CHD with depression was obtained by comparing the component target with the disease target analysis. Then, the component–target network between the targets of active components in XPF and CHD with depression was established using Cytoscape (Version 3.8.0) (Otasek et al., 2019).

### Topological Analysis and Pathway Enrichment Analysis

A network analyzer was used to calculate the topological analysis of the nodes of the component–target network. Nodes with degrees higher than the average number 4) were identified as the core targets, and were brought into the STRING database (https://string-db.org/) (Szklarczyk et al., 2017) to obtain the interacting proteins. Then, the PPI network was constructed using Cytoscape for visual analysis and, according to topological features (degree centrality), the major hub target was extracted. Further, the core targets were brought into the DAVID database (https://david.ncifcrf.gov) (Dennis et al., 2003), and Kyoto Encyclopedia of Genes and Genomes (KEGG) signaling pathway enrichment analysis and Gene ontology (GO) were performed to obtain the representative biological processes and pathways (cutoff at *p <* 0.05) of XPF in treating CHD with depression.

### Animal Model Preparation and Drug Treatments

Sixty-four male Sprague-Dawley rats (weighing 200 ± 20 g) were randomly divided into a normal control group (*n* = 8) and a depressive-like behavioral group (*n* = 56). Experimental rats (*n* = 56) were exposed to chronic unpredictable mild stress (CUMS) to induce depression. The regimen consisted of immobilization for 4 h; cage tilting at 45° for 12 h; continuous illumination for 24 h; clip the distal 1 cm rat tails with tongs for 3 min; deprivation of water for 24 h; noise stimulation for 30 min; damp animal bedding for 24 h; foot shock for 3 min; removal of animal bedding for 24 h; high-speed agitation for 10 min; deprivation of food 24 h; and forced cold swim stress for 6 min at 4°C. Each animal received two stressors randomly per day for a total of 28 days. The normal control group (*n* = 8) remained undisturbed. Subsequently, the CUMS-induced rats were injected with ISO (8 mg/kg) for five days to form the CHD model (Aa et al., 2019; Hu et al., 2020). The rat model for CHD with depression induced by CUMS and ISO was evaluated by measuring rat body weight change, open field test, sucrose preference test, and ST-segment of the electrocardiogram. Then, all CHD with depression rats were randomly divided into a model group (*n* = 15, 0.9% saline, IG), Ser + Met group (*n* = 8, sertraline 10 mg/kg + metoprolol 5 mg/kg, IG), Ser group (*n* = 8, sertraline 10 mg/kg, IG), Met group (*n* = 8, metoprolol 10 mg/kg, IG), high-dose XPF (XPF-H) group (*n* = 8, 32.7 g/kg, IG), equivalent to CH 3.13 g/kg, CX 2.5 g/kg, XF 2.5 g/kg, TX 1.88 g/kg, ZQ 3.13 g/kg, CP 3.13 g/kg, BX 3.13 g/kg, BH 2.08 g/kg, FL 3.13 g/kg, BZ 3.13 g/kg, ZS 3.13 g/kg, GC 3.13 g/kg, medium-dose XPF (XPF-M) group (*n* = 8, 16.35 g/kg, IG), equivalent to CH 1.65 g/kg, CX 1.25 g/kg, XF 1.25 g/kg, TX 0.94 g/kg, ZQ 1.65 g/kg, CP 1.65 g/kg, BX 1.65 g/kg, BH 1.04 g/kg, FL 1.65 g/kg, BZ 1.65 g/kg, ZS 1.65 g/kg, GC 0.94 g/kg, and low-dose XPF (XPF-L) group (*n* = 8, 8.175 g/kg, IG), equivalent to CH 0.78 g/kg, CX 0.62 g/kg, XF 0.63 g/kg, TX 0.47 g/kg, ZQ 0.78 g/kg, CP 0.78 g/kg, BX 0.78 g/kg, BH 0.52 g/kg, FL 0.78 g/kg, BZ 0.78 g/kg, ZS 0.78 g/kg, GC 0.47 g/kg. The control group received 0.9% saline via oral administration. The drugs were administered daily for three weeks, while the animals were exposed to CUMS, except for the control group. At 24 h after the last treatment, all rats were sacrificed, and the brain, hippocampus, and heart tissues were rapidly extracted from each rat, stored at −80°C until analysis. One part of the brain and heart tissues were placed into a flask containing 4% paraformaldehyde.

### TUNEL Staining

TUNEL staining was performed according to the manufacturer’s instructions, to determine hippocampus and myocardial apoptosis. The apoptotic cells showed red fluorescence and the nucleus showed blue fluorescence, six high-power fields were selected from each sample. All cells and positive stained cells were counted, the percentage of positively stained cells was apoptotic index (AI) (AI = number of apoptotic cells/total number of nucleated cells).

### ELISA Analysis

The level of 5-HT, Ang-Ⅱ, cAMP in hippocampus and myocardial were quantified by ELISA assay kit, according to the manufacturer's instruction. After color development, the absorbance was measured at 450 nm with fluorescence reader (THERMO USA).

### Western Blot Analysis

Hippocampus, hearts tissue proteins were mechanically homogenized in lysis buffer, centrifuged at 12,000 rpm for 10 min at 4 °C, collected the supernatant. BCA protein assay was used to determine protein concentrations. Equal concentrations of protein were resolved on 10% SDS-PAGE gels, and were transferred onto PVDF membranes. These membranes PVDF were soaked in TBST buffer with 5% non-fat skim milk for 2 h, and then These membranes PVDF were incubated in the primary antibodies (PKA, CREB, BDNF, Bcl-2, Bax, Cyt-c, caspase-3, and β-actin) overnight at 4°C. Subsequently, the membrane was washed for 5 times with TBST, followed by secondary antibodies incubated with horseradish peroxidase for 2 h at room temperature. After rewashing with TBST, the membranes were scanned on X-ray film by chemiluminescence reaction, used ImageJ software to analysis the band intensity.

### Statistical Analysis

Data were expressed as mean ± SD, Differences between groups were analyzed using a one-way analysis of variance (ANOVA), followed by Dunnett’s *t* test. All data were analyzed statistically using GraphPad Prism 8.0 (GraphPad Software, Inc., La Jolla, CA, USA), *p*-value < 0.05 was considered significant.

## Results

### Serum Pharmacochemistry Analysis of XPF by UPLC-Q-TOF/MS

**FIGURE 2:**
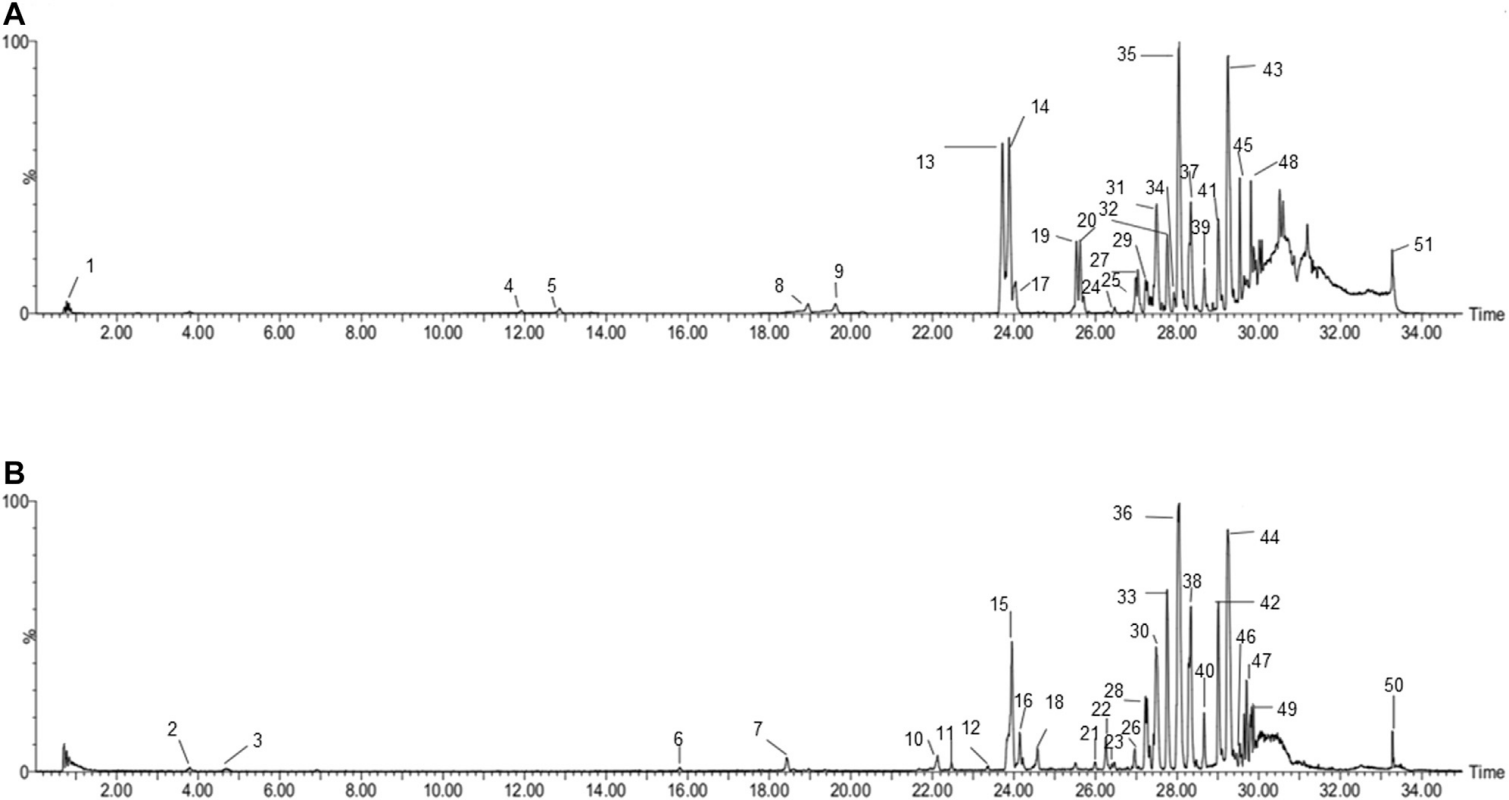
BPI chromatograms of XPF in serum sample in positive **(A)** and negative. **(B)** Ion modes determined by UPLC-Q-TOF/MS.

Based on the established UPLC-Q-TOF/MS method, 51 serum prototypes of the drugs were analyzed and identified **(** Table 1). These compounds can be roughly divided into three categories: phenolic acids, saponins, and flavonoids. Figure 2 shows the base peak chromatogram (BPC) of each typical sample in the mode of positive and negative ions, and peaks 1–51 are the original components entering the blood. These 51 compounds of XPF detected in the serum were determined to be the main active components and further selected to predict the targets and pathways using network analysis.

**TABLE 1:** Identification of absorbed components from XPF in serum of rats.

No	tR (min)	Name	Formula	Heoretical molecular weightigh/Da	[M + H]+		[M-H}-	
					Quasi-molecular ecular	Ppm	Quasi-moecular lecular	Ppm
1	1.42	Succinic acid	C4H6O4	117.0187			117.0185	2.8372
2	3.75	7-Methoxycoumarin	C10H8O3	177.0552	177.0557	2.8239		
3	5.44	Ferulic acid	C10H10O4	193.0501			193.0502	0.5698
4	5.86	Phenylalanine	C9H11NO2	166.0869	166.0865	2.4083		
5	12.48	Benzyl alcohol	C7H8O	131.0473	131.0475	1.5261		
6	15.95	Lonicerin	C27H30O15	593.1507	595.1671	1.1761	593.1488	3.237
7	18.42	Vanillic acid	C8H8O4	167.0344			167.0345	0.2334
8	18.90	Chrysophanol	C15H10O4	255.0658	255.066	0.7841		
9	19.64	Quercetin	C15H10O7	303.0505	303.0508	0.9899		
10	21.5	Liquiritoside	C21H22O9	417.1186	419.1338	1.1929	417.1193	1.6302
11	22.47	Rhoifolin	C27H30O14	577.1558	579.1721	3.6258	577.1557	0.1906
12	23.39	Protocatechuic acid	C7H6O4	153.0187			153.0182	3.8492
13	23.87	Acacetin	C16H12O5	285.0763	285.0753	3.6481		
14	23.87	Physcion	C16H12O5	285.0763	285.0764	0.2104		
15	23.98	Neoisoliquiritin	C21H22O9	417.1186			417.1176	2.4453
16	21.40	Naringin	C27H32O14	579.1714			579.1696	3.2288
17	21.70	Naringenin	C15H12O5	273.0763	273.077	2.5633		
18	24.54	Nobiletin	C21H22O8	403.1394	403.1399	1.2402		
19	25.57	Hesperetin	C16H14O6	303.0869	303.0875	1.9466		
20	25.64	Liquiritigenin	C15H12O4	257.0814	257.0811	1.1669		
21	26.06	Hesperidin	C28H34015	609.1820			609.1802	3.0204
22	26.24	Naringenine-7-rhamnosidoglucoside	C21H22O8	403.1394			403.1389	1.2403
23	26.49	Neoliquiritin	C21H22O9	417.1186			417.1177	2.2775
24	26.61	Formononetin	C16H12O4	269.0814	269.0816	0.6317		
25	27.02	Sinensetin	C20H20O7	373.1288	373.1274	3.7520		
26	27.05	Licochalcone B	C16H14O5	285.0763			285.0769	2.1047
27	27.15	Aloe-emodin	C15H10O5	271.0607	271.0615	2.9513		
28	237.34	Eugenol	C10H12O2	163.076			163.0755	3.3113
29	27.45	Emodin	C15H10O5	271.0607	271.0615	2.9513		
30	27.45	Spathulenol	C15H24O4	267.1597			267.1601	1.4972
31	27.54	Kaempferol	C15H10O6	287.0555	287.0571	5.2603		
32	27.78	Isorhamnetin	C16H12O7	317.0661	317.0652	3.0277		
33	27.81	Isoliquiritigenin	C15H12O4	255.0658			255.0663	1.9602
34	27.89	Angelicin	C11H6O3	187.0395	187.0389	3.2078		
35	28.01	Cerevisterol	C28H46O3	430.0325	430.0311	3.2555		
36	28.13	β-Amyrin acetate	C32H52O2	468.3969	468.3954	3.3732		
37	28.23	(−)-Hesperetin`	C16H14O6	303.0869	303.0867	0.6928		
38	28.45	Rosmarinic acid	C18H16O8	359.0767			359.0771	1.0304
39	28.52	Aesculetin	C9H6O4	179.0345	179.0339	3.5747		
40	28.81	Diosmin	C28H32O15	607.1663			607.1683	3.1622
41	29.13	Atractylenolide-1	C15H18O2	231.1385	231.1387	0.4759		
42	29.18	Saikosaponin A	C42H68O13	779.4584			779.4559	3.2587
43	29.21	4-Hydroxy-3-butylphthalideylphthalide	C12H14O3	207.1021	207.1026	2.0279		
44	29.29	Tangeretin (6CI)	C42H68O13	779.4584			779.4559	3.2587
45	29.53	α-Cyperone	C15H22O	219.175	219.1759	4.1063		
46	29.57	5,6,4′-trihydroxy-7,8,3′-trimethoxyflavonexy-7,8,3′-trimethoxyflavone	C18H16O8	359.0767			359.0771	1.0304
47	29.78	Menthyl benzoatee	C8H8O2	135.0446			135.0446	0.5183
48	29.83	β-Cyperol	C15H22O	219.175	219.1755	2.2812		
49	29.88	Tangeretin	C20H20O7	373.1288			373.1276	3.216
50	33.2	Natsudaidain	C20H20O7	417.1186	417.1188	0.4794	417.1193	1.6781
51	33.22	5,6,7,8-Tetramethoxy-2-(4-methoxyphenyl)-4-benzopyronethoxy-2-(4-methoxyphenyl)-4-benzopyrone	C20H20O7	373.1288	373.1279	2.4120		

### Component-Target Network Construction

**FIGURE 3:**
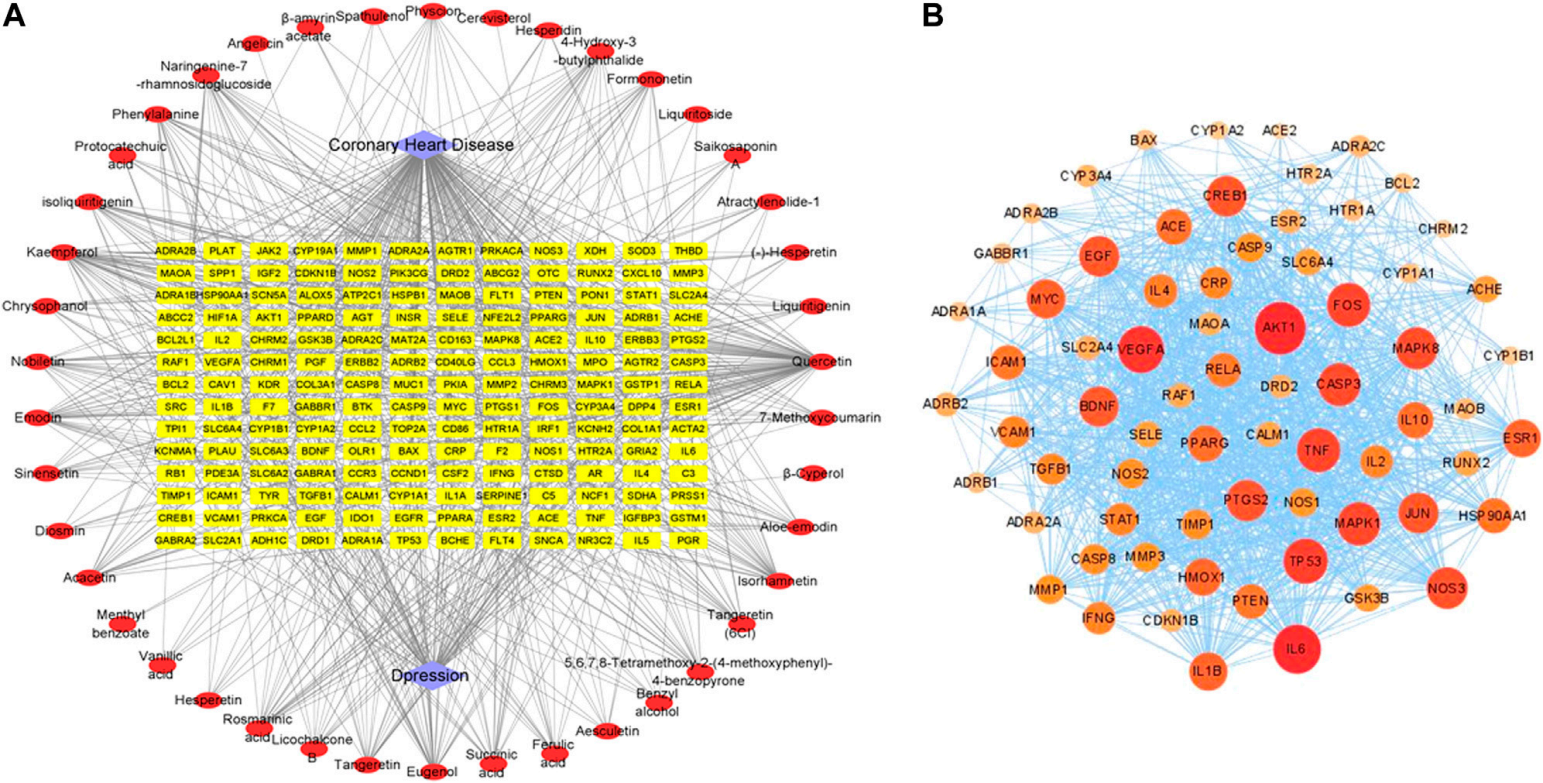
The networks associating with XPF in the treatment of CHD with depression. **(A)** The component-target network consisted of 42 components, 168 targets and 2 diseases including 212 nodes and 845 edges. **(B)** The PPI network of hub targets obtained from STRING database and constructed by Cytoscape.

In this work, 820 targets for the 51 (Nine of them had no therapeutic targets) components were explored by using TCMSP, Swiss Target Prediction, Drugbank, and Swiss Target Prediction as shown in Supplementary Table S1, and 1817 candidate targets of CHD and depression were obtained from Drugbank, OMIM, and DisGeNet databases as shown in Supplementary Table S2. Taking the intersection of component targets and candidate targets associated with CHD and depression, 168 consensus targets were generated as potential targets for XPF in treating CHD and depression, which were used to construct a component–target network using Cytoscape. As shown in Figure 3A, the network comprised 42 components, 168 targets, and two diseases, and included 212 nodes and 845 edges.

### Topological Analysis and Pathway Enrichment Analysis

**FIGURE 4:**
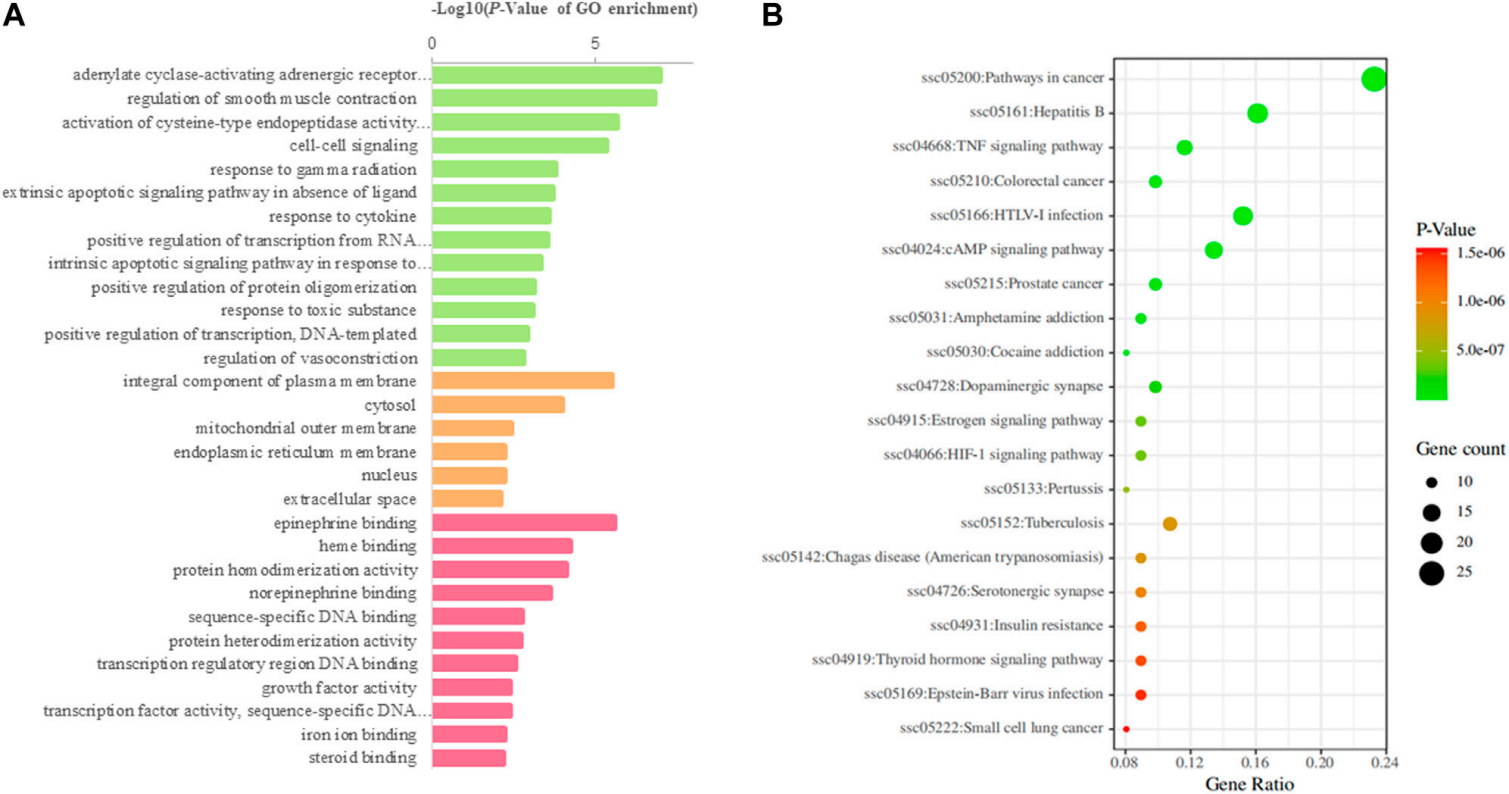
GO and KEGG analysis of the candidate targets. **(A)** Biological process enrichment of candidate targets. **(B)** KEGG pathwayenrichment of candidate targets.

Among the 168 consensus targets, 75 major targets with degrees higher than the average number 4) were considered as the major targets **(** Table 2). Ten components with degrees higher than 20 were identified as the main active components (Kaempferol and quercetin were not included because they are widely distributed and have many targets, but their pharmacological effects are weak) (Table 3). The 75 major targets were brought into the STRING database to obtain protein-protein interaction (PPI) predictions. The PPI network was constructed using Cytoscape, and hide disconnected nodes in the network (5 nodes). A network analyzer was used to calculate topological features (degree) of the 70 major targets (Figure 3B). Among these, 10 target genes (*ACE2*, *HTR1A*, *HTR2A*, *AKT1*, *PKIA*, *CREB1*, *BDNF*, *BCL2*, *BAX*, and *CASP3*), with higher degree were recognized as the major hub targets of XPF in treating CHD with depression. KEGG pathway enrichment and GO analyses of the 75 major targets were performed using the DAVID database. GO analysis showed 184 enriched processes (Supplementary Table S3), including 126 biological processes, 39 molecular functions, and 19 cellular components. The top 30 according to *p*-values are shown in Figure 4A. KEGG pathway enrichment showed 117 signaling pathways, as shown in Supplementary Table S4, and the top 20 pathways according to *p*-values are shown in Figure 4B.

**TABLE 2:** The topological parameters of 32 major targets.

Swiss prot	Genes/proteins	Description	Degree
P31749	AKT1	RAC-alpha serine/threonine-protein kinase	74
P05231	IL6	Interleukin-6	68
P15692	VEGFA	Vascular endothelial growth factor A	64
P04637	TP53	Cellular tumor antigen p53	61
P01100	FOS	Proto-oncogene c-Fos	59
P01375	TNF	Tumor necrosis factor	59
P28482	MAPK1	Mitogen-activated protein kinase 1	58
P42574	CASP3	Caspase-3	57
P45983	MAPK8	Mitogen-activated protein kinase 8	56
P29474	NOS3	Nitric oxide synthase	55
P35354	PTGS2	Prostaglandin G/H synthase 2	54
P16220	CREB1	Cyclic AMP-responsive element-binding protein 1	53
P01133	EGF	Pro-epidermal growth factor	53
P05412	JUN	Transcription factor AP-1	53
P23560	BDNF	Brain-derived neurotrophic factor	52
P01106	MYC	Myc proto-oncogene protein	50
P01584	IL1B	Interleukin-1 beta	49
P03372	ESR1	Estrogen receptor	48
P09601	HMOX1	Heme oxygenase 1	47
P37231	PPARG	Peroxisome proliferator-activated receptor gamma	45
P22301	IL10	Interleukin-10	45
P12821	ACE	Angiotensin-converting enzyme	44
P07900	HSP90AA1	Heat shock protein HSP 90-alpha	43
P05362	ICAM1	Intercellular adhesion molecule 1	42
P60484	PTEN	Phosphatidylinositol 3,4,5-trisphosphate 3-phosphatase and dual-specificity protein phosphatase PTEN	42
Q04206	RELA	Transcription factor p65	41
P05112	IL4	Interleukin-4	40
P42224	STAT1	Signal transducer and activator of transcription 1-alpha/beta	38
P01579	IFNG	Interferon gamma	38
P60568	IL2	Interleukin-2	38
P19320	VCAM1	Vascular cell adhesion protein 1	37
P02741	CRP	C-reactive protein	37

**TABLE 3:** The topological parameters of 20 main active components.

Compounds	Degree
Eugenol	28
Emodin	27
Isorhamnetin	25
Nobiletin	23
Isoliquiritigenin	22
Rosmarinic acid	22
4-Hydroxy-3 -butylphthalide	21
Acacetin	20

### Experimental Validation

#### Evaluation of Depressive-like Behaviors of Chronic Stress-Depressed Rats

The open field test can reflect the spatial exploration behavior of rats (Chen et al., 2019), and sucrose preference test could be the objective indicator of hedonic behavior (Gross and Pinhasov, 2016b). All rats were evaluated by open field test, sucrose preference test before and after the CUMS and ISO-induced process, then all CHD with depression rats were randomly divided into 7 groups. After three weeks of administration, open field test and sucrose preference test were performed again of all rats. As shown in Table 4, after CUMS and ISO-induced, compared with the control group the open-field test and sucrose preference scores were significantly reduced in the model group and each administration groups (*p <* 0.005). It was shown that the depression model was successfully established. After treatment for 21 days, compared with the model group, XPF administration significantly improve the open-field test and sucrose preference scores (p< 0.005).

**TABLE 4:** The comparison of the open field test scores and sucrose preference of rats in different groups.

Group	Open field test (scores)		Sucrose preference (%)	
	After CUMS and ISO-induced	After treatment	After CUMS and ISO-induced	After treatment
Control	160.17 ± 10.48	158.33 ± 12.4	91.69 ± 3.60	88.49 ± 2.91
Model	79.67 ± 6.80***	58.83 ± 3.06	58.89 ± 2.33***	55.03 ± 1.22
Ser + Met	82.83 ± 4.66***^△^	128.17 ± 8.4^###^	57.68 ± 2.79***^△^	81.17 ± 1.93^###^
Ser	77.5 ± 9.39***^△^	105.17 ± 7.9^###^	59.30 ± 2.69***^△^	76.01 ± 3.68^###^
Met	78.17 ± 6.94***^△^	96.67 ± 8.21^###^	56.51 ± 1.54***^△^	62.04 ± 1.40^###^
XPF-H	83.17 ± 2.63***^△^	148.50 ± 7.66^###^	58.25 ± 4.25***^△^	87.07 ± 2.16^###^
XPF-M	83.33 ± 0.13***^△^	122 ± 9.63^###^	57.39 ± 3.36***^△^	82.13 ± 1.52^###^
XPF-L	80.83 ± 7.98***^△^	109 ± 1^###^	57.76 ± 2.28***^△^	76.78 ± 1.72^###^

Values are expressed as the mean ± SD; *n* = 6–8 in each group. Compared with controll group after CUMS and ISO-induced.

***P < 0.005. Compared with model group after CUMS and ISO-induced, ^△^ *P* > 0.05. Compared with model group after treatment, ^###^ *P* < 0.005.

### Effects of XPF on Myocardial and Hippocampal Apoptosis

**FIGURE 5:**
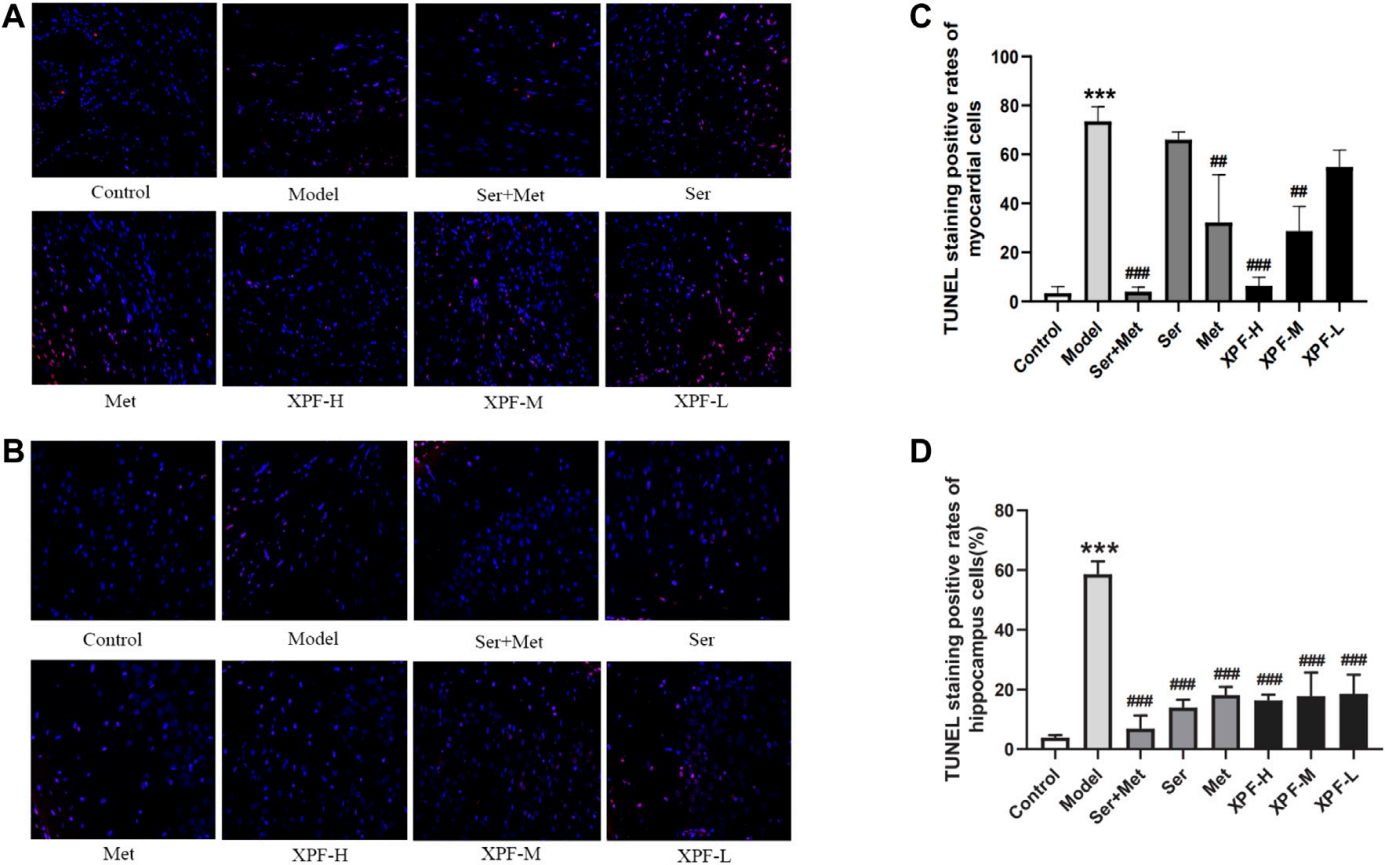
Anti-apoptosis effect of XPF on CUMS and ISO-induced rats (×400). **(A)** Representative images of myocardial apoptosis stained by TUNEL. **(B)** Representative images of hippocampal apoptosis stained by TUNEL. **(C)** Rate of myocardial apoptosis in each group. **(D)** Rate of hippocampal apoptosis in each group. Data wer presented as means ± SD (*n* = 3). ***p < 0.05 compared with the control group, ^##^ *p* < 0.01, ^##^ *p* < 0.005 compared with the model group.

Apoptosis can cause myocardial and hippocampal injury and dysfunction, which are the major mechanisms of CHD and depression. As shown in Figure 5, the myocardial and hippocampus of the CUMS and ISO-induced rats were injured. The effects of XPF on myocardial apoptosis are shown in Figure 5A. And the effects of XPF on hippocampal apoptosis are shown in Figure 5B. Compared with the control group, the myocardial and TUNEL-positive cells were significantly increased in the model group, as shown in Figure 5C (*p* < 0.005). The myocardial apoptotic index of the XPF-H and XPF-M groups was significantly lower than that of the model group (*p* < 0.01). Sertraline 10 mg/kg + metoprolol 5 mg/kg and metoprolol 10 mg/kg also significantly inhibited the apoptosis in the myocardia (Figure 5C) (*p* < 0.005). Sertraline (10 mg/kg) and low-dose XPF treatment did not alter myocardial apoptosis.

There were significant differences in the total apoptotic index in the hippocampus after XPF treatment for 21 days. In the model group, the apoptotic index significantly increased compared with the control group, as shown in Figure 5D. Furthermore, XPF administration significantly decreased the apoptotic index in the hippocampus. Additionally, sertraline and metoprolol also significantly inhibited apoptosis in the hippocampus (*p* < 0.005).

The results indicated that the brain and myocardial tissues of the rats were injured after CUMS and ISO-induced damage. It was shown that the CHD with depression model was successfully established, while the treatment of XPF can inhibit apoptosis of myocardial and brain tissue simultaneously.

According to key proteins with a higher degree in the PPI network and KEGG pathway enrichment analysis, it is suggested that the mechanism of XPF in the treatment of CHD with depression may be through the regulation of *ACE2*, *HTR1A*, *HTR2A*, *AKT1*, *PKIA*, *CREB1*, *BDNF*, *BCL2*, *BAX*, *CASP3*, cAMP signaling pathway, and the cell apoptosis process. As the second intracellular messenger, cAMP regulates the expression of downstream proteins and plays an important role in cell growth and apoptosis (Zhou et al., 2012). Bcl-2 and Bax, as targets downstream of CREB and BDNF, regulate apoptosis through the mitochondrial pathway (Mi et al., 2015; Tang et al., 2016). Recent studies have shown that the cAMP-PKA-CREB-BDNF signaling pathway is closely related to depression (Wang et al., 2017; Ye et al., 2017). Meanwhile, the related proteins in the cAMP-PKA-CREB-BDNF signaling pathway can also improve myocardial microcirculation, improve the ability of myocardial cells to resist ischemia and hypoxia, and effectively prevent the development and deterioration of CHD (Huang et al., 2016). To verify whether XPF works in treating CHD with depression by intervening in the cAMP-PKA-CREB-BDNF signaling pathway and cell apoptosis process, the expression of 5-HT, Ang-II, cAMP, PKA, CREB, BDNF, Bcl-2, Bax, Cyt-c, and caspase-3 in the myocardial and hippocampal tissues were detected.

#### 3.4.3 Effects of XPF on the Expression of Ang-II, 5-HT, and cAMP in Myocardial Tissues

**FIGURE 6:**
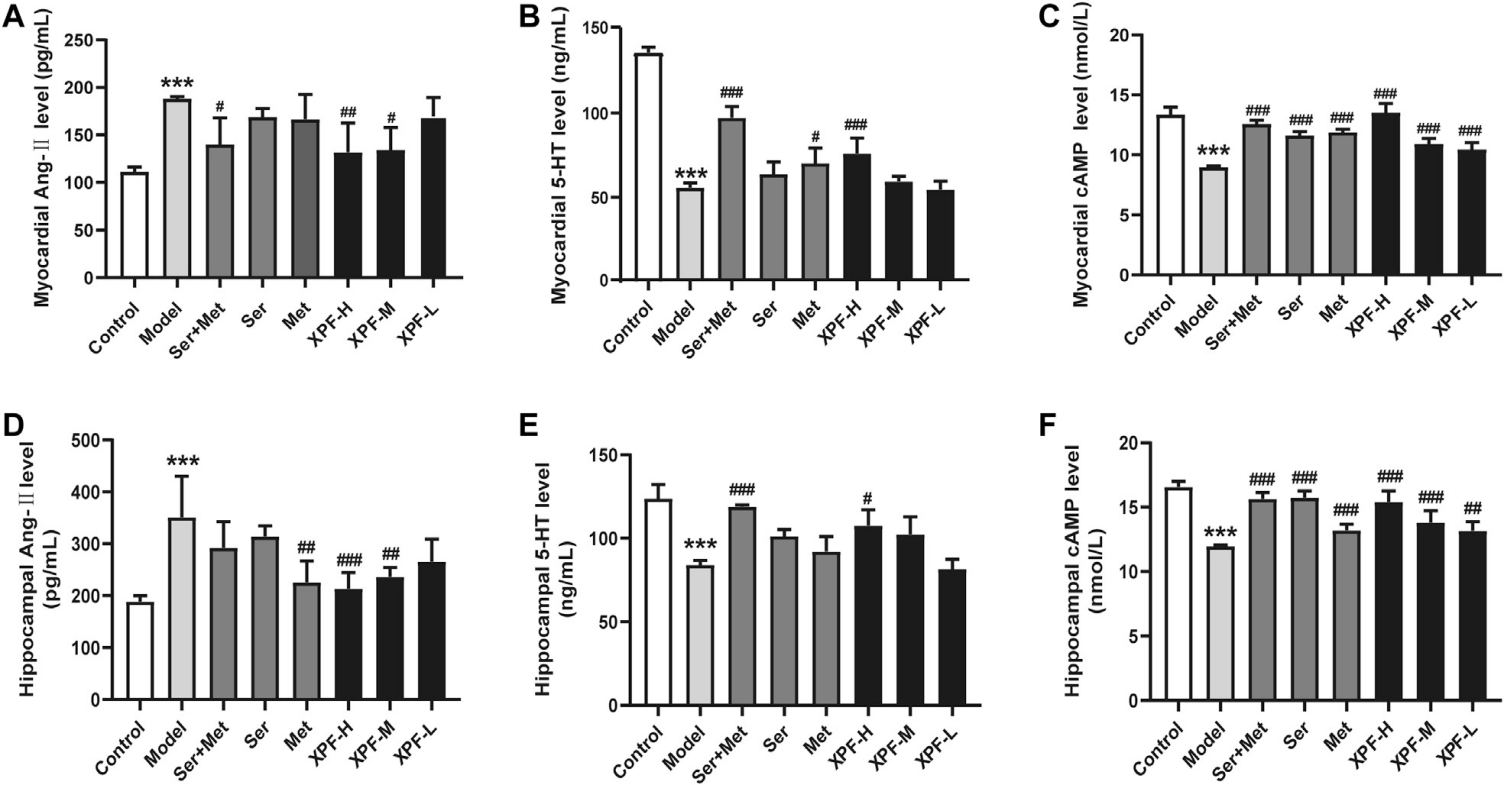
Experimental validation of key targets Ang-II, 5-HT, cAMP in rat myocardium and hippocampus induced by CUMS and ISO. **(A)** The levels of Ang-II in the myocardial were determined by ELISA. **(B)** The levels of 5-HT in the myocardial were determined by ELISA. **(C)** The levels of cAMP in the myocardial were determined by ELISA. **(D)** The levels of Ang-II in the hippocampus were determined by ELISA. **(E)** The levels of 5-HT in the hippocampus were determined by ELISA. **(F)** The levels of cAMP in the hippocampus were determined by ELISA. Data were presented as means ± SD (*n* = 3). ***p < 0.005 compared with the control group, ^##^ *p* < 0.05, ^##^ *p* < 0.01, ^###^ *p* < 0.005.

As shown in Figures 6A–C, it was noted that compared with the control group, the levels of Ang-II and 5-HT were significantly increased in the myocardial tissues in the model group. Compared with the control group, the expression of cAMP in the myocardial tissues was markedly lower in the model group (*p* < 0.005). After treatment for 21 days, high-dose XPF decreased the levels of Ang-II and 5-HT in the myocardium (*p* < 0.01). The treatments with middle-dose XPF solely decreased the levels of Ang-II (*p* < 0.05), whereas low-dose XPF had no effect on the levels of Ang-II and 5-HT in the myocardium. The levels of cAMP were significantly increased, in a dose-dependent manner, in the myocardium of the rats in the XPF-H, XPF-M, and XPF-L groups (*p* < 0.005).

#### 3.4.4 Effects of XPF on the Expression of Ang-II and 5-HT cAMP in the Hippocampus

As shown in Figures 6D–F, it was noted that, compared with the control group, the levels of Ang-II were significantly increased in the hippocampus of the model group. Conversely, the expression of 5-HT and cAMP in the hippocampus was markedly lower in the model group. After treatment for 21 days, the treatment with high-dose XPF and middle-dose XPF significantly decreased the levels of Ang-II (*p* < 0.005) and increased the levels of 5-HT and cAMP in the hippocampus (*p* < 0.05). Similarly, treatment with metoprolol markedly decreased the levels of Ang-II, and treatment with sertraline increased the levels of 5-HT and cAMP in the hippocampus (*p* < 0.05). However, low-dose XPF had no effect on the levels of Ang-II, 5-HT, and cAMP in the hippocampus.

#### Effects of XPF on the Expression of PKA, CREB, and BDNF on Myocardial Tissue

**FIGURE 7:**
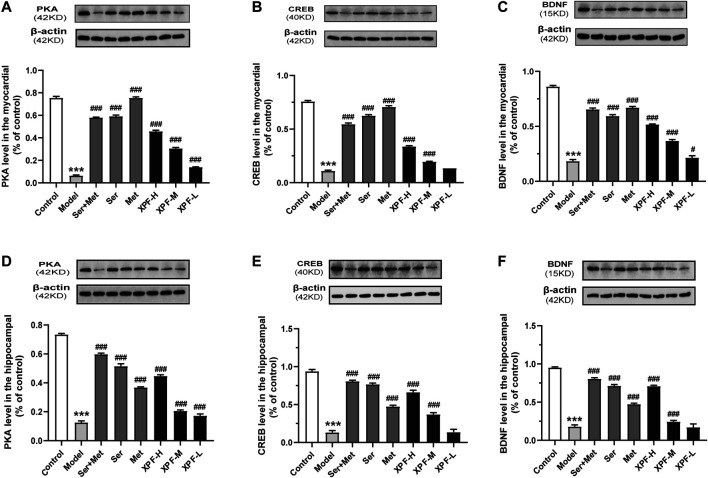
Experimental validation of key targets PKA, CREB, BDNF in rat myocardium and hippocampus induced by CUMS and ISO. **(A)** Quantitative analysis of PKA expression in the myocardial. **(B)** Quantitative analysis of CREB expression in the myocardial. **(C)** Quantitative analysis of BDNF expression in the hippocampal. **(D)** Quantitative analysis of PKA expression in the hippocampal. **(E)** Quantitative analysis of CREB expression in the hippocampal. **(F)** Quantitative analysis of CREB expression in the hippocampal. Data were presented as means ± SD (*n* = 3). ***p < 0.005 compared with the control group, ^##^ *p* < 0.01, ^##^ *p* < 0.005.

As presented in Figures 7A–C, the results of the WB analysis indicated that the expression of PKA, CREB, and BDNF in the myocardium in the model group was significantly lower than that of the control group (*p* < 0.005). XPF could upregulate the expression of PKA, CREB, and BDNF compared with the model group (*p* < 0.05).

#### Effects of XPF on the Expression of PKA, CREB, and BDNF in the Hippocampus

As shown in Figures 7D–F, the PKA, CREB, and BDNF protein levels of the model group were significantly lower than those of the control group. High-dose XPF and middle-dose XPF was able to upregulate the expression of PKA, CREB, and BDNF compared with the model group (*p* < 0.005). However, low-dose XPF had no effect on the levels of CREB and BDNF in the hippocampus.

#### Effects of XPF on the Expression of Bcl-2, Bax, Cyt-C, and Caspase-3 in the Myocardium

**FIGURE 8:**
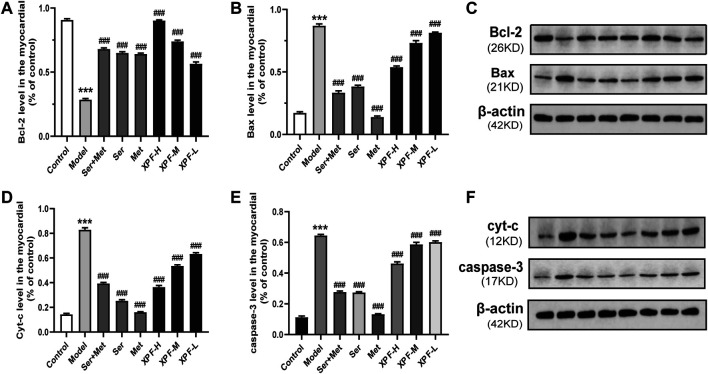
Experimental validation of key targets Bcl-2, BAX, Cyt-c, caspase-3 in rat myocardium induced by CUMS and ISO. **(A)** Quantitative analysis of Bcl-2 expression in the myocardial. **(B)** Quantitative analysis of BAX expression in the myocardial. **(C)** Western blot analysis of Bcl-2 and BAX protein. **(D)** Quantitative analysis of Cyt-c expression in the myocardial. **(E)** Quantitative analysis of caspase-3 expression in the myocardial. **(F)** Western blot analysis of Cyt-c and caspase-3 protein. Data were presented as means ± SD (*n* = 3). ***p < 0.005 compared with the control group, ^###^ *p* < 0.005 compared with the model group.

As shown in Figure 8 and Table 5, compared with the control group, the levels of Bax, Cyt-c, and caspase-3 were significantly increased in the model group, although Bcl-2 levels significantly decreased. Treatment with XPF significantly increased the levels of Bcl-2 and decreased the levels of Bax, Cyt-c, and caspase-3 (*p* < 0.005), in a dose dependent manner.

**TABLE 5:** Comparison of gray values of Bcl-2, Bax, Cyt-C and Caspase-3 in myocardium of rats in different groups.

Group	Bal-2/β-actin	Bax/β-actin	Cyt-c/β-actin	Caspase/β-actin
Control	0.9080 ± 0.0083	0.1728 ± 0.0084	0.1437 ± 0.0077	0.1139 ± 0.0071
Model	0.2864 ± 0.0076***	0.8701 ± 0.0143***	0.8300 ± 0.0159***	0.6454 ± 0.0072***
Ser + Met	0.6814 ± 0.0072^###^	0.3348 ± 0.0142^###^	0.3945 ± 0.0075^###^	0.2768 ± 0.0067^###^
Ser	0.6505 ± 0.0086^###^	0.3850 ± 0.0085^###^	0.2534 ± 0.0085^###^	0.2743 ± 0.0044^###^
Met	0.6419 ± 0.0067^###^	0.1408 ± 0.0070^###^	0.1615 ± 0.0029^###^	0.1328 ± 0.0029^###^
XPF-H	0.9032 ± 0.0035^###^	0.5394 ± 0.0084^###^	0.3657 ± 0.0113^###^	0.4630 ± 0.0104^###^
XPF-M	0.7402 ± 0.0083^###^	0.7331 ± 0.0173^###^	0.5366 ± 0.0082^###^	0.5879 ± 0.0126^###^
XPF-L	0.5665 ± 0.0130^###^	0.8143 ± 0.0053^###^	0.6337 ± 0.0096^###^	0.6024 ± 0.0069^###^

Values are expressed as the mean ± SD; *n* = 3 in each group. Compared with control.

***P < 0.005, Compared with model.

^###^ *P* < 0.005.

#### Effects of XPF on the Expression of Bcl-2, Bax, Cyt-C, and Caspase-3 in the Hippocampus

**FIGURE 9:**
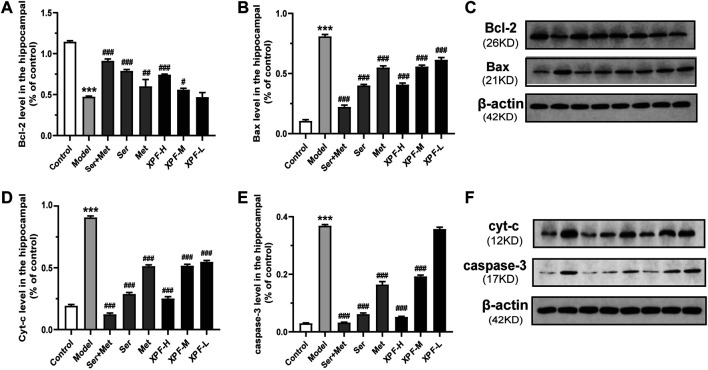
Experimental validation of key targets Bcl-2, BAX, Cyt-c, caspase-3 in rat hippocampus induced by CUMS and ISO. **(A)** Quantitative analysis of Bcl-2 expression in the hippocampal. **(B)** Quantitative analysis of BAX expression in the hippocampal. **(C)** Western blot analysis of Bcl-2 and BAX protein. **(D)** Quantitative analysis of Cyt-c expression in the hippocampal. **(E)** Quantitative analysis of caspase-3 expression in the hippocampal. **(F)** Western blot analysis of Cyt-c and caspase-3 protein. Data were presented as means ± SD (*n* = 3). ***p < 0.005 compared with the control group, ^###^ *p* < 0.005 compared with the model group.

As shown in Figure 9 and Table 6, the results of the WB analysis indicated that the expression of Bax, Cyt-c, and caspase-3 was significantly increased in the model group, while Bcl-2 levels were significantly decreased. After treatment for 21 days, high-dose XPF and middle-dose XPF significantly decreased the levels of Bax, Cyt-c, and caspase-3 (*p* < 0.005). High-dose, and middle-dose XPF also increased the levels of Bcl-2 (*p* < 0.05), in a dose-dependent manner. In contrast, low-dose XPF only inhibited the expression of Bax and Cyt-c (*p* < 0.005), but had no effect on the expression of Bcl-2 and caspase-3.

**TABLE 6:** Comparison of gray values of Bcl-2, Bax, Cyt-C and Caspase-3 in hippocampus of rats in different groups.

Group	Bal-2/β-actin	Bax/β-actin	Cyt-c/β-actin	Caspase/β-actin
Control	1.1464 ± 0.0112	0.1064 ± 0.0098	0.1937 ± 0.0103	0.0301 ± 0.0009
Model	0.4744 ± 0.0081***	0.8098 ± 0.0144***	0.9091 ± 0.0106***	0.3689 ± 0.0040***
Ser + Met	0.9136 ± 0.0217^###^	0.2226 ± 0.0147^###^	0.1248 ± 0.0090^###^	0.0323 ± 0.0015^###^
Ser	0.7909 ± 0.0143^###^	0.4004 ± 0.0085^###^	0.2885 ± 0.0122^###^	0.0620 ± 0.0038^###^
Met	0.6014 ± 0.0832^##^	0.5492 ± 0.0142^###^	0.5143 ± 0.0094^###^	0.1646 ± 0.0103^###^
XPF-H	0.7445 ± 0.0061^###^	0.4084 ± 0.0119^###^	0.2529 ± 0.0131^###^	0.0521 ± 0.0017^###^
XPF-M	0.5623 ± 0.0151^#^	0.5590 ± 0.0104^###^	0.5180 ± 0.0106^###^	0.1928 ± 0.0043^###^
XPF-L	0.4703 ± 0.0535	0.6148 ± 0.0181^###^	0.5496 ± 0.0097^###^	0.3575 ± 0.0063

Values are expressed as the mean ± SD; *n* = 3 in each group. Compared with control.

***P < 0.005, Compared with model, ^#^ *P* < 0.05, ^##^ *P* < 0.01, ^###^ *P* < 0.005.

## Discussion

XPF, characterized by multiple components and targets, has been shown to have a relatively satisfactory therapeutic effect in treating CHD with depression. XPF can improve myocardial blood supply and mental state of patients at the same time, which reflects the overall concept of traditional Chinese medicine (Hou et al., 2017). Through this study, we found that XPF could protect the myocardium and hippocampus in CUMS and ISO-induced CHD in depression rats, it shows that XPF has the advantages and characteristics of treating the heart and brain at the same time. However, it is difficult to accurately identify and understand the active compounds and mechanisms of XPF in treating CHD with depression solely by using conventional pharmacological methods. Therefore, new research approaches to reveal the interaction between components of TCM and biological system networks are urgently required. Here, an integrated strategy was established by combining serum pharmacochemistry and network pharmacology to comprehensively and systematically investigate the components and mechanisms of XPF in treating CHD with depression, and validated in experiments.

In this study, 51 compounds were detected in rat serum after oral administration of XPF using UPLC-Q-TOF/MS technology. The identified constituents, mainly phenolic acids, saponins, and flavonoids, were determined to be the main active components of XPF. Among them, phenolic acids, such as rosmarinic acid and eugenol, can inhibit vascular smooth muscle cell proliferation and have a cardioprotective effect (Liu Y et al., 2018). Rosmarinic acid can treat neurodegenerative diseases via anti-neuroinflammatory activity (Wei et al., 2018). Furthermore, saponins, such as saikosaponin A, are the major bioactive component extracted from Radix Bupleuri (Chen et al., 2018; Liu R et al., 2018) and have anti-inflammatory and antioxidant pharmacological activities, as well as a good therapeutic effect on many diseases of the central nervous system and cardiovascular system (Guo et al., 2020). Flavonoids, such as hesperidin and hesperetin, can inhibit inflammatory reactions and oxidative stress, and protect nerve cells and vascular endothelium (Hwang et al., 2012; Van Rymenant et al., 2017).

Moreover, the component–target network between the targets of absorbed components in the serum of XPF and CHD with depression was built using Cytoscape, followed by topological parameters and PPI analysis. As a result, 8 components (eugenol, emodin, isorhamnetin, nobiletin, isoliquiritigenin, rosmarinic acid, 4-Hydroxy-3-butylphthalide, and acacetin), with higher degree centrality were determined to be the active ingredients of XPF, and 10 hub targets, including (ACE2, HTR1A, HTR2A, AKT1, PKIA, CREB1, BDNF, BCL2, BAX, CASP3, cAMP) were determined as hub targets of XPF in treating CHD with depression. GO analysis and KEGG pathway enrichment analysis showed that XPF may regulate the cAMP signaling and apoptosis pathways by interfering with the expression of Ang-II, 5-HT, cAMP, PKA, CREB, BDNF, Bcl-2, Bax, Cyt-c, and caspase-3.

In this study, CUMS and ISO-induced CHD in a rat model of depression was used to explore the mechanism of XPF in treating CHD with depression. These rats showed a large number of apoptotic cells in myocardial and hippocampal tissues compared with the control group, which indicated that the CHD with depression model was successfully established. Sertraline and metoprolol were used as positive control drugs to treat CHD in rats with depression. Sertraline can increase the content of 5-hydroxytryptamine (5-HT) in the brain and exert antidepressant-like effects (Sun et al., 2020). Metoprolol is a beta-blocker commonly used in the clinic (Podlesnikar et al., 2019; Qin et al., 2020). Studies have shown that metoprolol could reduce the oxygen consumption of the myocardium and reduce the risk of cardiovascular events (Assimon et al., 2018). However, co-administration of sertraline and metoprolol increases the blood concentration of metoprolol. Therefore, we adjusted the dose of metoprolol in the sertraline and metoprolol groups in this study (Bahar et al., 2018).

Under these experimental conditions, XPF could decrease Ang-II content in the circulation and central system, inhibit 5-HT level in peripheral circulation, and increase 5-HT content in the central nervous system. Meanwhile, XPF could upregulate the expression levels of cAMP, PKA, CREB, and BDNF both in the myocardium and hippocampus, which matched the predicted results of the network pharmacological analysis. Interestingly, sertraline regulated the cAMP, PKA, CREB, and BDNF levels in the hippocampus, but had a limited effect on the myocardium. Metoprolol can regulate the cAMP, PKA, CREB, and BDNF expression in myocardial, but it has a limited effect on the central nervous system. XPF treatment not only activated the cAMP signal pathway, but also further inhibited apoptosis and protected the myocardium and hippocampus. The cAMP signaling pathway can increase the expression of Bcl-2 and inhibit the activation of caspase-3 by regulating the expression of CREB and BDNF proteins (Mo et al., 2020; Yang et al., 2020). In this study, XPF rectified the injury of the hippocampus and myocardium caused by CHD in depressed rats, significantly decreasing the hippocampal and myocardial levels of Bax, Cyt-c, caspase-3, and increased Bcl-2 expression, which suggests that XPF may inhibit hippocampal and myocardial apoptosis, improve heart function and nerve function, and exert antidepressant and anti-myocardial ischemia effects.

Chronic stressful life events can stimulate the renin-angiotensin-aldosterone system (RAAS). Evidence has shown that Ang-II levels are significantly higher in patients with CHD and depression. Ang-II is metabolized into Ang-(1–7), which directly interferes with the intestinal uptake of tryptophan and affects the metabolism of 5-HT (Klempin et al., 2018; W et al., 2020). The decrease of 5-HT levels in the central nervous system leads to depression or other mental disorders (Jiao et al., 2019; Wenxiu et al., 2020). 5-HT metabolic disorder inhibits the cAMP signaling pathway (Louiset et al., 2017). The cAMP signaling pathway was observed in both CHD and depression patients (Amare et al., 2017; Wang and Mao, 2019; Sandrini et al., 2020).

Activated cAMP and PKA can phosphorylate the Ca^2+^channel on the cell membrane, promote calcium flow in cardiomyocytes, promote excitation contraction coupling, enhance myocardial contractility, improve the anti-ischemic effects of cardiomyocytes, and produce protective effects on cardiomyocytes (Inserte et al., 2004; Zhang et al., 2010). At the same time, cAMP and PKA are also widely distributed in the nervous system, which can guide the regeneration of neurons, improve the plasticity of synapses, and play a role in protecting the nervous system (Zhang M et al., 2018). CREB is involved in a wide range of neural plasticity processes, including neuronal survival, neuroprotection, neurogenesis, synaptic plasticity, and regulation of emotional expression (Liu et al., 2016; Tang et al., 2020). BDNF is a classical downstream target gene of CREB, which can nourish nerves and promote the differentiation, increment, and regeneration of neurons (Wang et al., 2019). BDNF also has a modulatory role in cardiovascular function, is involved in angiogenesis maintenance of endothelial integrity (Kaess et al., 2015; Jin et al., 2018), repairing myocardial microcirculation, and maintaining myocardial cell function after myocardial injury (Lee et al., 2017). Low expression of CREB and BDNF causes Bax overexpression, Bcl-2 expression is inhibited, and the promotion of the release of apoptosis factors such as cytochrome c (Cyt-c) into the cytoplasm and increased caspase-3. Caspase-3, the final executive factor of apoptosis, leads to apoptosis (Xu et al., 2018). Bcl-2 can form a heterodimer with Bax to inhibit its function, Bcl-2 can also inhibit the activation of caspase-3, and inhibit apoptosis (Abu et al., 2018). Meng (Meng and Wang, 2013) have found that Baishile (BSL) capsule could significantly improve the CUMS rats with depression-like behavior; K252a could block the cAMP signaling pathway to antagonize the antidepressant effect of BSL capsule. Jiang (Jiang et al., 2017) have found that acupuncture could improve depressive-like behaviors via PKA/CREB signaling pathway, H89 could block the PKA/CREB signaling pathway to antagonize the antidepressant effect of acupuncture. Mao (Mao and Zhang, 2014) have found H89 could block the PKA/CREB signaling pathway to antagonize the myocardial protective effect of Danlou tablets. Therefore, enhanced expression of related proteins in the cAMP signaling pathway and inhibition of the cell apoptosis process contributes to the alleviation of angina pectoris, chest pain, and depression symptoms. Essentially, it promotes protection of the cardiovascular and nervous system against coronary heart disease with depression.

The 8 active components (eugenol, emodin, isorhamnetin, nobiletin, isoliquiritigenin, rosmarinic acid, 4-Hydroxy-3-butylphthalide, and acacetin) in XPF have been reported to activate the cAMP signaling pathway of CHD and depression. Eugenol can reverse oxidative stress, inhibit of caspase-3 activity, and it has an anti-myocardial ischemia effect. Emodin opposes CUMS-induced depressive-like behavior in rats by upregulating the levels of hippocampal BDNF (Choudhary et al., 2006; Liu et al., 2017). Isorhamnetin has a positive effect on H/R-induced injury by reducing caspase-3 and attenuating oxidative stress in H9c2 cardiomyocytes (Zhao et al., 2018). Nobiletin and acacetin exerted antidepressant-like effects by increasing the level of 5-HT in the hippocampus (Yi et al., 2011). Isoliquiritigenin has been shown to have neuroprotective effects by increasing the level of cAMP in peripheral nerves (Shindo et al., 1992). Rosmarinic acid can regulate the expression of BDNF in the hippocampus (Makhathini et al., 2018), and inhibit angiotensin-converting enzyme activity (Ferreira et al., 2018).

Increasing evidence has shown a reciprocal causation relationship between CHD and depression. Regulating psychopathy can significantly improve the quality of life of patients with CHD and reduce the incidence of acute cardiovascular events (Kuhlmann et al., 2019). Recently, TCM has attracted increasing attention in treating cardiovascular and psychological diseases because of its holistic approach and satisfactory clinical efficacy. Through this study, we found that XPF could protect the myocardium and hippocampus in CUMS and ISO-induced CHD in depression rats by regulating the cAMP signal cascade. Furthermore, XPF can inhibit cell apoptosis, improve heart function, reduce neuropathy, improve nerve function, and exert an antidepressant and antimyocardial ischemia effect. These results preliminarily show the material basis and mechanism of XPF in the treatment of CHD with depression.

## Conclusion

In this study, we proposed an integrative systems pharmacology strategy to illustrate the mechanism of XPF in treating CHD with depression by combining serum pharmacochemistry, network analysis, and experimental validation. First, we identified 51 ingredients in rat serum after oral administration of XPF, which was used for the construction and analysis of the component–target network between the targets of absorbed components in serum and CHD with depression. As a result, 10 components with higher degree centrality were determined to be the active ingredients of XPF, and 10 hub genes, including *ACE2*, *HTR1A*, *HTR2A*, *AKT1*, *PKIA*, *CREB1*, *BDNF*, *BCL2*, *BAX*, *CASP3*, and *cAMP* were determined as targets of XPF in treating CHD with depression. Furthermore, the hub targets (AngⅡ, 5-HT, cAMP, PKA, CREB, BDNF, Bcl-2, Bax, Cyt-c, and caspase-3) predicted by network pharmacology analysis were validated. We confirmed the myocardial protective and neuroprotective effects of XPF in treating CHD with depression, which was associated with its activation of the cAMP signaling pathway and inhibition of myocardium and hippocampus apoptosis. In conclusion, these results indicate that the integrated pharmacology strategy provides an efficient approach for exploring the pharmacological mechanisms of TCM.

## Data Availability

The raw data supporting the conclusions of this article will be made available by the authors, without undue reservation.
