# Supplementary material for: An Integrative Pharmacology-Based Strategy to Uncover the Mechanism of Xiong-Pi-Fang in Treating Coronary Heart Disease with Depression
Source: Front Pharmacol. 2021 Apr 1;12:590602. doi: 10.3389/fphar.2021.590602 (PMC8048422; doi:10.3389/fphar.2021.590602)
Supplement: Supplementary file 1 [file table1.docx]

**TABLE S1 ׀** Putative targets of 51 components in XPF.

| **Compounds** | **Mol ID** | **Description** | **Genes/proteins** | **Swiss prot** |
| --- | --- | --- | --- | --- |
| Succinic acid | MOL000346 | Aspartate aminotransferase, cytoplasmic | GOT1 | P20309 |
|  |  | Aldose reductase | AKR1B1 | A0A024R7A8 |
|  |  | Proto-oncogene tyrosine-protein kinase SRC | SRC | P61925 |
|  |  | Cathepsin D | CTSD | P50416 |
|  |  | M-phase inducer phosphatase 2 | CDC25B | P25054 |
|  |  | Cholinesterase | BCHE | P25054 |
|  |  | Alcohol dehydrogenase 1C | ADH1C | A0A087WU81 |
|  |  | Alcohol dehydrogenase 1A | ADH1A | P07327 |
|  |  | Aspartate aminotransferase | GIG18 | P20309 |
|  |  | Monomeric sarcosine oxidase | PIPOX | P16615 |
|  |  | Aspartate aminotransferase, mitochondrial | GOT2 | P20309 |
|  |  | S-adenosylmethionine synthetase isoform type-1 | MAT1A | Q9NQ11 |
|  |  | Triosephosphate isomerase | TPI1 | P61925 |
|  |  | NAD-dependent malic enzyme, mitochondrial | ME2 | Q9NQ11 |
|  |  | NADP-dependent malic enzyme, mitochondrial | ME3 | Q9NQ11 |
|  |  | Trypsin-3 | PRSS3 | P16615 |
|  |  | Nitric-oxide synthase, brain | NOS1 | Q9NQ11 |
|  |  | Ornithine aminotransferase, mitochondrial | OAT | P16615 |
|  |  | mRNA of Protein-tyrosine phosphatase, non-receptor type 1 | PTPN1 | P16615 |
|  |  | Gamma-aminobutyric acid receptor subunit alpha-1 | GABRA1 | P20309 |
|  |  | mRNA of PKA Catalytic Subunit C-alpha | PRKACA | P16615 |
|  |  | Adenylosuccinate synthetase | ADSS1 | G3V232 |
|  |  | Glutamate dehydrogenase 1, mitochondrial | GLUD1 | P20309 |
|  |  | Ornithine carbamoyltransferase, mitochondrial | OTC | P16615 |
|  |  | Serine--pyruvate aminotransferase | AGXT | A2V838 |
|  |  | Succinate dehydrogenase [ubiquinone] flavoprotein subunit, mitochondrial | SDHA | P16615 |
|  |  | S-adenosylmethionine synthetase isoform type-2 | MAT2A | Q9NQ11 |
|  |  | Prolyl 3-hydroxylase 1 | P3H1 | P16615 |
|  |  | Prolyl 3-hydroxylase 3 | P3H3 | P16615 |
|  |  | Succinyl-CoA:3-ketoacid-coenzyme A transferase 2, mitochondrial | OXCT2 | P16615 |
|  |  | Mitochondrial dicarboxylate carrier | SLC25A10 | P61925 |
|  |  | Prolyl 4-hydroxylase subunit alpha-2 | P4HA2 | P16615 |
|  |  | Caspase-3 | CASP3 | P25054 |
| 7-Methoxycoumarin | MOL004617 | Prostaglandin G/H synthase 1 | PTGS1 | P16615 |
|  |  | Prostaglandin G/H synthase 2 | PTGS2 | P16615 |
|  |  | Alpha-2A adrenergic receptor | ADRA2A | P08913 |
|  |  | Sodium-dependent dopamine transporter | SLC6A3 | P61925 |
|  |  | Sodium-dependent serotonin transporter | SLC6A4 | P61925 |
|  |  | Leukotriene A-4 hydrolase | LTA4H | Q9NQ11 |
|  |  | Amine oxidase [flavin-containing] B | MAOB | Q9NQ11 |
|  |  | Amine oxidase [flavin-containing] A | MAOA | Q9NQ11 |
|  |  | mRNA of PKA Catalytic Subunit C-alpha | PRKACA | P16615 |
|  |  | Muscarinic acetylcholine receptor M1 | CHRM1 | P25054 |
|  |  | Alpha-1A adrenergic receptor | ADRA1A | B0ZBD3 |
|  |  | Dipeptidyl peptidase IV | DPP4 | P50416 |
|  |  | cAMP-dependent protein kinase inhibitor alpha | PKIA | P16615 |
| Ferulic acid | MOL002665 | Prostaglandin G/H synthase 1 | PTGS1 | P16615 |
|  |  | Muscarinic acetylcholine receptor M1 | CHRM1 | P25054 |
|  |  | Beta-1 adrenergic receptor | ADRB1 | P08588 |
|  |  | Prostaglandin G/H synthase 2 | PTGS2 | P16615 |
|  |  | Nitric-oxide synthase, endothelial | NOS3 | Q9NQ11 |
|  |  | Alpha-2A adrenergic receptor | ADRA2A | P08913 |
|  |  | Sodium-dependent noradrenaline transporter | SLC6A2 | P61925 |
|  |  | Alpha-1A adrenergic receptor | ADRA1A | B0ZBD3 |
|  |  | Muscarinic acetylcholine receptor M2 | CHRM2 | P25054 |
|  |  | Alpha-2B adrenergic receptor | ADRA2B | L8E8D5 |
|  |  | Beta-2 adrenergic receptor | ADRB2 | A2N4T7 |
|  |  | Beta-lactamase | BQ8482 | P25054 |
|  |  | Leukotriene A-4 hydrolase | LTA4H | Q9NQ11 |
|  |  | Amine oxidase [flavin-containing] B | MAOB | Q9NQ11 |
|  |  | Amine oxidase [flavin-containing] A | MAOA | Q9NQ11 |
|  |  | cAMP-dependent protein kinase inhibitor alpha | PKIA | P16615 |
| Phenylalanine | MOL000041 | Prostaglandin G/H synthase 1 | PTGS1 | P16615 |
|  |  | Muscarinic acetylcholine receptor M1 | CHRM1 | P25054 |
|  |  | Beta-1 adrenergic receptor | ADRB1 | P08588 |
|  |  | Sodium channel protein type 5 subunit alpha | SCN5A | P16615 |
|  |  | Prostaglandin G/H synthase 2 | PTGS2 | P16615 |
|  |  | Nitric-oxide synthase, endothelial | NOS3 | Q9NQ11 |
|  |  | Sodium-dependent noradrenaline transporter | SLC6A2 | P61925 |
|  |  | Alpha-1A adrenergic receptor | ADRA1A | B0ZBD3 |
|  |  | Sodium-dependent dopamine transporter | SLC6A3 | P61925 |
|  |  | Dipeptidyl peptidase IV | DPP4 | P50416 |
|  |  | Beta-lactamase | BQ8482 | P25054 |
|  |  | Amine oxidase [flavin-containing] B | MAOB | Q9NQ11 |
|  |  | Lysozyme | LYZA | Q9NQ11 |
|  |  | Amine oxidase [flavin-containing] A | MAOA | Q9NQ11 |
|  |  | Chymotrypsinogen B | CTRB1 | P50416 |
|  |  | Dopamine D1 receptor | DRD1 | P50416 |
|  |  | Alpha-2B adrenergic receptor | ADRA2B | L8E8D5 |
| Benzyl alcohol | MOL001335 | Alcohol dehydrogenase 1A | ADH1A | P07327 |
|  |  | Nicotinate-nucleotide--dimethylbenzimidazole phosphoribosyltransferase | ACE2 | Q9BYF1 |
|  |  | Lysozyme | LYZA | Q9NQ11 |
|  |  | Sodium channel protein type 5 subunit alpha | SCN5A | P16615 |
|  |  | Prostaglandin G/H synthase 2 | PTGS2 | P16615 |
|  |  | Heat shock protein HSP 90 | HSP90AA1 | Q9NQ11 |
|  |  | Calmodulin | CALM1 | P25054 |
|  |  | Prostaglandin G/H synthase 1 | PTGS1 | P16615 |
|  |  | CGMP-inhibited 3',5'-cyclic phosphodiesterase A | PDE3A | P16615 |
| Vanillic acid | MOL000114 | Prostaglandin G/H synthase 2 | PTGS2 | P16615 |
|  |  | Amine oxidase [flavin-containing] B | MAOB | Q9NQ11 |
|  |  | Amine oxidase [flavin-containing] A | MAOA | Q9NQ11 |
|  |  | Alpha-2A adrenergic receptor | ADRA2A | P08913 |
|  |  | Prostaglandin G/H synthase 1 | PTGS1 | P16615 |
|  |  | Lysozyme | LYZD2 | Q9NQ11 |
|  |  | Nitric oxide synthase, endothelial | NOS3 | Q9NQ11 |
|  |  | 5,6-dihydroxyindole-2-carboxylic acid oxidase | TYRP1 | P61925 |
| Chrysophanol | MOL001729 | Prostaglandin G/H synthase 1 | PTGS1 | P16615 |
|  |  | Prostaglandin G/H synthase 2 | PTGS2 | P16615 |
|  |  | Heat shock protein HSP 90 | HSP90AA1 | Q9NQ11 |
|  |  | Nuclear receptor coactivator 2 | NCOA2 | Q9NQ11 |
|  |  | Calmodulin | CALM1 | P25054 |
|  |  | Sodium channel protein type 5 subunit alpha | SCN5A | P16615 |
|  |  | CGMP-inhibited 3',5'-cyclic phosphodiesterase A | PDE3A | P16615 |
|  |  | Gamma-aminobutyric acid receptor subunit alpha-1 | GABRA1 | P20309 |
|  |  | Phosphatidylinositol-4,5-bisphosphate 3-kinase catalytic subunit, gamma isoform | PIK3CG | P16615 |
|  |  | mRNA of PKA Catalytic Subunit C-alpha | PRKACA | P16615 |
|  |  | Ig gamma-1 chain C region | IGHG1 | Q9NQ11 |
| Quercetin | MOL000098 | Prostaglandin G/H synthase 1 | PTGS1 | P16615 |
|  |  | Peroxisome proliferator activated receptor gamma | PPARG | P16615 |
|  |  | Prostaglandin G/H synthase 2 | PTGS2 | P16615 |
|  |  | Heat shock protein HSP 90 | HSP90AA1 | Q9NQ11 |
|  |  | Phosphatidylinositol-4,5-bisphosphate 3-kinase catalytic subunit, gamma isoform | PIK3CG | P16615 |
|  |  | Nuclear receptor coactivator 2 | NCOA2 | Q9NQ11 |
|  |  | Dipeptidyl peptidase IV | DPP4 | P50416 |
|  |  | Trypsin-1 | PRSS1 | P16615 |
|  |  | DNA topoisomerase II | TOP2A | P61925 |
|  |  | Potassium voltage-gated channel subfamily H member 2 | KCNH2 | Q9NQ11 |
|  |  | Sodium channel protein type 5 subunit alpha | SCN5A | P16615 |
|  |  | Stromelysin-1 | MMP3 | Q9NQ11 |
|  |  | mRNA of PKA Catalytic Subunit C-alpha | PRKACA | P16615 |
|  |  | Coagulation factor VII | F7 | P20309 |
|  |  | Nitric-oxide synthase, endothelial | NOS3 | Q9NQ11 |
|  |  | Retinoic acid receptor RXR-alpha | RXRA | P16615 |
|  |  | Gamma-aminobutyric acid receptor subunit alpha-1 | GABRA1 | P20309 |
|  |  | Amine oxidase [flavin-containing] B | MAOB | Q9NQ11 |
|  |  | Transcription factor p65 | RELA | P16615 |
|  |  | Epidermal growth factor receptor | EGFR | P50416 |
|  |  | Vascular endothelial growth factor A | VEGFA | P61925 |
|  |  | G1/S-specific cyclin-D1 | CCND1 | P25054 |
|  |  | BCL2 | BCL2 | P25054 |
|  |  | Bcl-2-like protein 1 | BCL2L1 | P25054 |
|  |  | Proto-oncogene c-Fos | FOS | P20309 |
|  |  | Cyclin-dependent kinase inhibitor 1 | CDKN1B | P25054 |
|  |  | Eukaryotic translation initiation factor 6 | EIF6 | P50416 |
|  |  | Apoptosis regulator BAX | BAX | P25054 |
|  |  | Caspase-9 | CASP9 | P25054 |
|  |  | Urokinase-type plasminogen activator | PLAU | P16615 |
|  |  | 72 kDa type IV collagenase | MMP2 | Q9NQ11 |
|  |  | Matrix metalloproteinase-9 | MMP9 | Q9NQ11 |
|  |  | Mitogen-activated protein kinase 1 | MAPK1 | Q9NQ11 |
|  |  | Interleukin-10 | IL10 | Q9NQ11 |
|  |  | Pro-epidermal growth factor | EGF | P50416 |
|  |  | Retinoblastoma-associated protein | RB1 | P16615 |
|  |  | Tumor necrosis factor | TNF | P61925 |
|  |  | Transcription factor AP-1 | JUN | Q9NQ11 |
|  |  | Interleukin-6 | IL6 | Q9NQ11 |
|  |  | Activator of 90 kDa heat shock protein ATPase homolog 1 | AHSA1 | G3V3W9 |
|  |  | Caspase-3 | CASP3 | P25054 |
|  |  | Cellular tumor antigen p53 | TP53 | P61925 |
|  |  | ETS domain-containing protein Elk-1 | ELK1 | P50416 |
|  |  | NF-kappa-B inhibitor alpha | NFKBIA | Q9NQ11 |
|  |  | NADPH--cytochrome P450 reductase | POR | P16615 |
|  |  | Ornithine decarboxylase | ODC1 | P16615 |
|  |  | Xanthine dehydrogenase/oxidase | XDH | P61925 |
|  |  | Caspase-8 | CASP8 | P25054 |
|  |  | DNA topoisomerase 1 | TOP1 | P61925 |
|  |  | RAF proto-oncogene serine/threonine-protein kinase | RAF1 | P16615 |
|  |  | Superoxide dismutase [Cu-Zn] | SOD3 | P61925 |
|  |  | Protein kinase C alpha type | PRKCA | P16615 |
|  |  | Interstitial collagenase | MMP1 | Q9NQ11 |
|  |  | Hypoxia-inducible factor 1-alpha | HIF1A | Q9NQ11 |
|  |  | Signal transducer and activator of transcription 1-alpha/beta | STAT1 | P61925 |
|  |  | Protein CBFA2T1 | RUNX1T1 | P16615 |
|  |  | 78 kDa glucose-regulated protein | HSPA5 | Q9NQ11 |
|  |  | Receptor tyrosine-protein kinase erbB-2 | ERBB2 | P50416 |
|  |  | Peroxisome proliferator-activated receptor gamma | PPARG | P16615 |
|  |  | Heme oxygenase 1 | HMOX1 | Q9NQ11 |
|  |  | Cytochrome P450 3A4 | CYP3A4 | P50416 |
|  |  | Cytochrome P450 1A2 | CYP1A2 | P50416 |
|  |  | Caveolin-1 | CAV1 | P25054 |
|  |  | Myc proto-oncogene protein | MYC | Q9NQ11 |
|  |  | Gap junction alpha-1 protein | GJA1 | P20309 |
|  |  | Cytochrome P450 1A1 | CYP1A1 | P50416 |
|  |  | Intercellular adhesion molecule 1 | ICAM1 | Q9NQ11 |
|  |  | Interleukin-1 beta | IL1B | Q9NQ11 |
|  |  | C-C motif chemokine 2 | CCL2 | P25054 |
|  |  | E-selectin | SELE | P16615 |
|  |  | Vascular cell adhesion protein 1 | VCAM1 | P61925 |
|  |  | Prostaglandin E2 receptor EP3 subtype | PTGER3 | P16615 |
|  |  | Interleukin-8 | IL8 | Q9NQ11 |
|  |  | Protein kinase C beta type | PRKCB | P16615 |
|  |  | Baculoviral IAP repeat-containing protein 5 | BIRC5 | P25054 |
|  |  | Dual oxidase 2 | DUOX2 | P50416 |
|  |  | Nitric oxide synthase, endothelial | NOS3 | Q9NQ11 |
|  |  | Heat shock protein beta-1 | HSPB1 | Q9NQ11 |
|  |  | Transforming growth factor beta-1 | TGFB1 | P61925 |
|  |  | Estrogen sulfotransferase | SULT1E1 | P61925 |
|  |  | Maltase-glucoamylase, intestinal | MGAM | Q9NQ11 |
|  |  | Interleukin-2 | IL2 | Q9NQ11 |
|  |  | Nuclear receptor subfamily 1 group I member 2 | NR1I2 | P16615 |
|  |  | Cytochrome P450 1B1 | CYP1B1 | P50416 |
|  |  | G2/mitotic-specific cyclin-B1 | CCNB1 | P25054 |
|  |  | Tissue-type plasminogen activator | PLAT | P16615 |
|  |  | Thrombomodulin | THBD | P61925 |
|  |  | Plasminogen activator inhibitor 1 | SERPINE1 | P16615 |
|  |  | Collagen alpha-1(I) chain | COL1A1 | Q00526 |
|  |  | Interferon gamma | IFNG | Q9NQ11 |
|  |  | Phosphatidylinositol-3,4,5-trisphosphate 3-phosphatase and dual-specificity protein phosphatase PTEN | PTEN | P16615 |
|  |  | Interleukin-1 alpha | IL1A | Q9NQ11 |
|  |  | Myeloperoxidase | MPO | Q9NQ11 |
|  |  | DNA topoisomerase 2-alpha | TOP2A | P61925 |
|  |  | Neutrophil cytosol factor 1 | NCF1 | Q9NQ11 |
|  |  | ATP-binding cassette sub-family G member 2 | ABCG2 | A1LUE4 |
|  |  | Hyaluronan synthase 2 | HAS2 | Q9NQ11 |
|  |  | Glutathione S-transferase P | GSTP1 | Q9NQ11 |
|  |  | Nuclear factor erythroid 2-related factor 2 | NFE2L2 | Q9NQ11 |
|  |  | NAD(P)H dehydrogenase [quinone] 1 | NQO1 | P16615 |
|  |  | Poly [ADP-ribose] polymerase 1 | PARP1 | P16615 |
|  |  | 26S proteasome non-ATPase regulatory subunit 3 | PSMD3 | P16615 |
|  |  | Solute carrier family 2, facilitated glucose transporter member 4 | SLC2A4 | P61925 |
|  |  | Collagen alpha-1(III) chain | COL3A1 | Q00526 |
|  |  | C-X-C motif chemokine 11 | CXCL11 | P50416 |
|  |  | C-X-C motif chemokine 2 | CXCL2 | P50416 |
|  |  | DDB1- and CUL4-associated factor 5 | DCAF5 | P50416 |
|  |  | Nuclear receptor subfamily 1 group I member 3 | NR1I3 | P16615 |
|  |  | Serine/threonine-protein kinase Chk2 | CHEK2 | P25054 |
|  |  | Insulin receptor | INSR | Q9NQ11 |
|  |  | Claudin-4 | CLDN4 | Q00526 |
|  |  | Peroxisome proliferator-activated receptor alpha | PPARA | P16615 |
|  |  | Peroxisome proliferator-activated receptor delta | PPARD | P16615 |
|  |  | Heat shock factor protein 1 | HSF1 | Q9NQ11 |
|  |  | C-reactive protein | CRP | P50416 |
|  |  | C-X-C motif chemokine 10 | CXCL10 | P50416 |
|  |  | Inhibitor of nuclear factor kappa-B kinase subunit alpha | CHUK | P25054 |
|  |  | Osteopontin | SPP1 | P61925 |
|  |  | Runt-related transcription factor 2 | RUNX2 | P16615 |
|  |  | Ras association domain-containing protein 1 | RASSF1 | P16615 |
|  |  | Transcription factor E2F1 | E2F1 | P50416 |
|  |  | Transcription factor E2F2 | E2F2 | P50416 |
|  |  | Prostatic acid phosphatase | ACPP | A0PJ86 |
|  |  | Cathepsin D | CTSD | P50416 |
|  |  | Insulin-like growth factor-binding protein 3 | IGFBP3 | Q9NQ11 |
|  |  | Insulin-like growth factor II | IGF2 | Q9NQ11 |
|  |  | CD40 ligand | CD40LG | P25054 |
|  |  | Interferon regulatory factor 1 | IRF1 | Q9NQ11 |
|  |  | Receptor tyrosine-protein kinase erbB-3 | ERBB3 | P50416 |
|  |  | Serum paraoxonase/arylesterase 1 | PON1 | P16615 |
|  |  | Type I iodothyronine deiodinase | DIO1 | P50416 |
|  |  | Procollagen C-endopeptidase enhancer 1 | PCOLCE | P16615 |
|  |  | Puromycin-sensitive aminopeptidase | NPEPPS | P16615 |
|  |  | Hexokinase-2 | HK2 | Q9NQ11 |
|  |  | Homeobox protein Nkx-3.1 | NKX3-1 | Q9NQ11 |
|  |  | Ras GTPase-activating protein 1 | RASA1 | P16615 |
|  |  | Glutathione S-transferase Mu 1 | GSTM1 | Q9NQ11 |
|  |  | Glutathione S-transferase Mu 2 | GSTM2 | Q9NQ11 |
| Liquiritoside | MOL004903 | Coagulation factor VII | F7 | P20309 |
|  |  | Calmodulin | CAMK1 | P25054 |
|  |  | Prostaglandin G/H synthase 2 | PTGS2 | P16615 |
|  |  | Superoxide dismutase [Cu-Zn] | SOD3 | P61925 |
| Acacetin | MOL001689 | Nitric oxide synthase, inducible | NOS2 | Q9NQ11 |
|  |  | Prostaglandin G/H synthase 1 | PTGS1 | P16615 |
|  |  | Prostaglandin G/H synthase 2 | PTGS2 | P16615 |
|  |  | Dipeptidyl peptidase IV | DPP4 | P50416 |
|  |  | Heat shock protein HSP 90 | HSP90AA1 | Q9NQ11 |
|  |  | Cell division protein kinase 2 | CDK2 | P25054 |
|  |  | mRNA of PKA Catalytic Subunit C-alpha | PRKACA | P16615 |
|  |  | Trypsin-1 | PRSS1 | P16615 |
|  |  | Nuclear receptor coactivator 2 | NCOA2 | Q9NQ11 |
|  |  | Nuclear receptor coactivator 1 | NCOA1 | Q9NQ11 |
|  |  | Calmodulin | CALM1 | P25054 |
|  |  | Phosphatidylinositol-4,5-bisphosphate 3-kinase catalytic subunit, gamma isoform | PIK3CG | P16615 |
|  |  | Serine/threonine-protein kinase Chk1 | CHEK1 | P25054 |
|  |  | CGMP-inhibited 3',5'-cyclic phosphodiesterase A | PDE3A | P16615 |
|  |  | Transcription factor p65 | RELA | P16615 |
|  |  | Apoptosis regulator Bcl-2 | BCL2 | P25054 |
|  |  | Cyclin-dependent kinase inhibitor 1 | CDKN1B | P25054 |
|  |  | Apoptosis regulator BAX | BAX | P25054 |
|  |  | Caspase-3 | CASP3 | P25054 |
|  |  | Cellular tumor antigen p53 | TP53 | P61925 |
|  |  | Caspase-8 | CASP8 | P25054 |
|  |  | Fatty acid synthase | FASN | P20309 |
|  |  | Tumor necrosis factor ligand superfamily member 6 | TNF | P61925 |
|  |  | Cytochrome P450 19A1 | CYP19A1 | P50416 |
| Physcion | MOL000476 | Prostaglandin G/H synthase 1 | PTGS1 | P16615 |
|  |  | Sodium channel protein type 5 subunit alpha | SCN5A | P16615 |
|  |  | Prostaglandin G/H synthase 2 | PTGS2 | P16615 |
|  |  | Nitric-oxide synthase, endothelial | NOS3 | Q9NQ11 |
|  |  | Coagulation factor VII | F7 | P20309 |
|  |  | DNA topoisomerase II | TOP2A | P61925 |
|  |  | Heat shock protein HSP 90 | HSP90AA1 | Q9NQ11 |
|  |  | Phosphatidylinositol-4,5-bisphosphate 3-kinase catalytic subunit, gamma isoform | PIK3CG | P16615 |
|  |  | Beta-lactamase | BQ8482 | P25054 |
|  |  | Ig gamma-1 chain C region | IGHG1 | Q9NQ11 |
|  |  | Nuclear receptor coactivator 2 | NCOA2 | Q9NQ11 |
|  |  | Nuclear receptor coactivator 1 | NCOA1 | Q9NQ11 |
|  |  | cAMP-dependent protein kinase inhibitor alpha | PKIA | P16615 |
|  |  | Calmodulin | CALM1 | P25054 |
|  |  | mRNA of PKA Catalytic Subunit C-alpha | PRKACA | P16615 |
| Physcion | MOL000476 | Retinoic acid receptor RXR-alpha | RXRA | P16615 |
| Nobiletin | MOL005828 | Prostaglandin G/H synthase 1 | PTGS1 | P16615 |
|  |  | Potassium voltage-gated channel subfamily H member 2 | KCNH2 | Q9NQ11 |
|  |  | Estrogen receptor | ESR | P20309 |
|  |  | Peroxisome proliferator activated receptor gamma | PPARG | P16615 |
|  |  | Prostaglandin G/H synthase 2 | PTGS2 | P16615 |
|  |  | Coagulation factor VII | F7 | P20309 |
|  |  | mRNA of Protein-tyrosine phosphatase, non-receptor type 1 | PTPN1 | P16615 |
|  |  | DNA topoisomerase II | TOP2A | P61925 |
|  |  | Estrogen receptor beta | ESR2 | P20309 |
|  |  | Dipeptidyl peptidase IV | DPP4 | P50416 |
|  |  | Heat shock protein HSP 90 | HSP90AA1 | Q9NQ11 |
|  |  | Serine/threonine-protein kinase Chk1 | CHEK1 | P25054 |
|  |  | Trypsin-1 | PRSS1 | P16615 |
|  |  | Nuclear receptor coactivator 2 | NCOA2 | Q9NQ11 |
|  |  | Calcium-activated potassium channel subunit alpha 1 | KCNMA1 | Q9NQ11 |
|  |  | Calmodulin | CALM1 | P25054 |
|  |  | Glycogen synthase kinase-3 beta | GSK3B | P49841 |
|  |  | Sodium channel protein type 5 subunit alpha | SCN5A | P16615 |
|  |  | Apoptosis regulator Bcl-2 | BCL2 | P25054 |
|  |  | Apoptosis regulator BAX | BAX | P25054 |
|  |  | Caspase-9 | CASP9 | P25054 |
|  |  | Matrix metalloproteinase-9 | MMP9 | Q9NQ11 |
|  |  | Transcription factor AP-1 | JUN | Q9NQ11 |
|  |  | Cellular tumor antigen p53 | TP53 | P61925 |
|  |  | Mitogen-activated protein kinase 8 | MAPK8 | Q9NQ11 |
|  |  | Metalloproteinase inhibitor 1 | TIMP1 | P61925 |
|  |  | Peroxisome proliferator-activated receptor gamma | PPARG | P16615 |
|  |  | Cyclic AMP-responsive element-binding protein 1 | CREB1 | P50416 |
|  |  | Cytosolic phospholipase A2 | PLA2G4A | P16615 |
|  |  | Scavenger receptor cysteine-rich type 1 protein M130 | CD163 | P25054 |
|  |  | Ephrin type-B receptor 2 | EPHB2 | P50416 |
| Hesperetin | MOL002341 | Prostaglandin G/H synthase 1 | PTGS1 | P16615 |
|  |  | Sodium channel protein type 5 subunit alpha | SCN5A | P16615 |
|  |  | Prostaglandin G/H synthase 2 | PTGS2 | P16615 |
|  |  | Heat shock protein HSP 90 | HSP90AA1 | Q9NQ11 |
|  |  | Phosphatidylinositol-4,5-bisphosphate 3-kinase catalytic subunit, gamma isoform | PIK3CG | P16615 |
|  |  | mRNA of PKA Catalytic Subunit C-alpha | PRKACA | P16615 |
|  |  | Nuclear receptor coactivator 2 | NCOA2 | Q9NQ11 |
|  |  | Nuclear receptor coactivator 1 | NCOA1 | Q9NQ11 |
|  |  | Acetylcholinesterase | ACHE | [P22303](https://www.uniprot.org/uniprot/P22303) |
|  |  | Androgen receptor | AR | P10275 |
|  |  | Calmodulin | CALM1 | P25054 |
| Liquiritigenin | MOL001792 | Prostaglandin G/H synthase 1 | PTGS1 | P16615 |
|  |  | Estrogen receptor | ESR1 | P20309 |
|  |  | Prostaglandin G/H synthase 2 | PTGS2 | P16615 |
|  |  | Retinoic acid receptor RXR-alpha | RXRA | P16615 |
|  |  | Beta-2 adrenergic receptor | ADRB2 | A2N4T7 |
|  |  | Heat shock protein HSP 90 | HSP90AA1 | Q9NQ11 |
|  |  | Phosphatidylinositol-4,5-bisphosphate 3-kinase catalytic subunit, gamma isoform | PIK3CG | P16615 |
|  |  | mRNA of PKA Catalytic Subunit C-alpha | PRKACA | P16615 |
|  |  | Beta-lactamase | BQ8482 | P25054 |
|  |  | Amine oxidase [flavin-containing] B | MAOB | Q9NQ11 |
|  |  | Sodium-dependent serotonin transporter | SLC6A4 | P61925 |
|  |  | cAMP-dependent protein kinase inhibitor alpha | PKIA | P16615 |
| Hesperidin | MOL007930 | Apoptosis regulator BAX | BAX | P25054 |
|  |  | Caspase-3 | CASP3 | P25054 |
|  |  | Prostaglandin G/H synthase 2 | PTGS2 | P16615 |
|  | | Intercellular adhesion molecule 1 | ICAM1 | Q9NQ11 |
|  | | Vascular cell adhesion protein 1 | VCAM1 | P61925 |
|  |  | T-lymphoma invasion and metastasis-inducing protein 2 | TIAM2 | P61925 |
| Naringenine-7-rhamnosidoglucoside | MOL005828 | Prostaglandin G/H synthase 1 | PTGS1 | P16615 |
|  |  | Potassium voltage-gated channel subfamily H member 2 | KCNH2 | Q9NQ11 |
|  |  | Estrogen receptor | ESR | P20309 |
|  |  | Peroxisome proliferator activated receptor gamma | PPARG | P16615 |
|  |  | Prostaglandin G/H synthase 2 | PTGS2 | P16615 |
|  |  | Coagulation factor VII | F7 | P20309 |
|  |  | mRNA of Protein-tyrosine phosphatase, non-receptor type 1 | PTPN1 | P16615 |
|  |  | DNA topoisomerase II | TOP2A | P61925 |
|  |  | Estrogen receptor beta | ESR2 | P20309 |
|  |  | Dipeptidyl peptidase IV | DPP4 | P50416 |
|  |  | Heat shock protein HSP 90 | HSP90AA1 | Q9NQ11 |
|  |  | Serine/threonine-protein kinase Chk1 | CHEK1 | P25054 |
|  |  | Trypsin-1 | PRSS1 | P16615 |
|  |  | Nuclear receptor coactivator 2 | NCOA2 | Q9NQ11 |
|  |  | Calcium-activated potassium channel subunit alpha 1 | KCNMA1 | Q9NQ11 |
|  |  | Calmodulin | CALM1 | P25054 |
|  |  | Glycogen synthase kinase-3 beta | GSK3B | P49841 |
|  |  | Sodium channel protein type 5 subunit alpha | SCN5A | P16615 |
|  |  | Apoptosis regulator Bcl-2 | BCL2 | P25054 |
|  |  | Apoptosis regulator BAX | BAX | P25054 |
|  |  | Caspase-9 | CASP9 | P25054 |
|  |  | Matrix metalloproteinase-9 | MMP9 | Q9NQ11 |
|  |  | Transcription factor AP-1 | JUN | Q9NQ11 |
|  |  | Cellular tumor antigen p53 | TP53 | P61925 |
|  |  | Mitogen-activated protein kinase 8 | MAPK8 | Q9NQ11 |
|  |  | Metalloproteinase inhibitor 1 | TIMP1 | P61925 |
|  |  | Peroxisome proliferator-activated receptor gamma | PPARG | P16615 |
|  |  | Cyclic AMP-responsive element-binding protein 1 | CREB1 | P50416 |
|  |  | Cytosolic phospholipase A2 | PLA2G4A | P16615 |
|  |  | Scavenger receptor cysteine-rich type 1 protein M130 | CD163 | P25054 |
|  |  | Ephrin type-B receptor 2 | EPHB2 | P50416 |
| Formononetin | MOL010586 | Nitric oxide synthase, inducible | NOS2 | Q9NQ11 |
|  |  | Prostaglandin G/H synthase 1 | PTGS1 | P16615 |
|  |  | Dopamine D1 receptor | DRD1 | P50416 |
|  |  | Muscarinic acetylcholine receptor M1 | CHRM1 | P25054 |
|  |  | Estrogen receptor | ESR1 | P20309 |
|  |  | Peroxisome proliferator activated receptor gamma | PPARG | P16615 |
|  |  | Prostaglandin G/H synthase 2 | PTGS2 | P16615 |
|  |  | Retinoic acid receptor RXR-alpha | RXRA | P16615 |
|  |  | Sodium-dependent dopamine transporter | SLC6A3 | P61925 |
|  |  | Beta-2 adrenergic receptor | ADRB2 | A2N4T7 |
|  |  | Sodium-dependent serotonin transporter | SLC6A4 | P61925 |
|  |  | Estrogen receptor beta | ESR2 | P20309 |
|  |  | Dipeptidyl peptidase IV | DPP4 | P50416 |
|  |  | Mitogen-activated protein kinase 14 | MAPK14 | Q9NQ11 |
|  |  | Glycogen synthase kinase-3 beta | GSK3B | P49841 |
|  |  | Heat shock protein HSP 90 | HSP90AA1 | Q9NQ11 |
|  |  | Cell division protein kinase 2 | CDK2 | P25054 |
|  |  | Amine oxidase [flavin-containing] B | MAOB | Q9NQ11 |
|  |  | mRNA of PKA Catalytic Subunit C-alpha | PRKACA | P16615 |
|  |  | Trypsin-1 | PRSS1 | P16615 |
|  |  | Proto-oncogene serine/threonine-protein kinase Pim-1 | PIM1 | P16615 |
|  |  | Cyclin-A2 | CCNA2 | P25054 |
|  |  | cAMP-dependent protein kinase inhibitor alpha | PKIA | P16615 |
| Sinensetin | MOL001803 | Nitric oxide synthase, inducible | NOS2 | Q9NQ11 |
|  |  | Potassium voltage-gated channel subfamily H member 2 | KCNH2 | Q9NQ11 |
|  |  | Sodium channel protein type 5 subunit alpha | SCN5A | P16615 |
|  |  | Prostaglandin G/H synthase 2 | PTGS2 | P16615 |
|  |  | Coagulation factor VII | F7 | P20309 |
|  |  | Beta-2 adrenergic receptor | ADRB2 | A2N4T7 |
|  |  | DNA topoisomerase II | TOP2A | P61925 |
|  |  | Estrogen receptor beta | ESR2 | P20309 |
|  |  | Dipeptidyl peptidase IV | DPP4 | P50416 |
|  |  | Heat shock protein HSP 90 | HSP90AA1 | Q9NQ11 |
|  |  | Serine/threonine-protein kinase Chk1 | CHEK1 | P25054 |
|  |  | Trypsin-1 | PRSS1 | P16615 |
|  |  | Nuclear receptor coactivator 2 | NCOA2 | Q9NQ11 |
|  |  | Nuclear receptor coactivator 1 | NCOA1 | Q9NQ11 |
|  |  | Calmodulin | CAMK1 | P25054 |
|  |  | Prostaglandin G/H synthase 1 | PTGS1 | P16615 |
| Licochalcone B | MOL004841 | Nitric oxide synthase, inducible | NOS2 | Q9NQ11 |
|  |  | Prostaglandin G/H synthase 1 | PTGS1 | P16615 |
|  |  | Estrogen receptor | ESR1 | P20309 |
|  |  | Peroxisome proliferator activated receptor gamma | PPARG | P16615 |
|  |  | Prostaglandin G/H synthase 2 | PTGS2 | P16615 |
|  |  | Carbonic anhydrase II | CA2 | P25054 |
|  |  | CGMP-inhibited 3',5'-cyclic phosphodiesterase A | PDE3A | P16615 |
|  |  | Beta-2 adrenergic receptor | ADRB2 | A2N4T7 |
|  |  | Estrogen receptor beta | ESR2 | P20309 |
|  |  | Mitogen-activated protein kinase 14 | MAPK14 | Q9NQ11 |
|  |  | Glycogen synthase kinase-3 beta | GSK3B | P49841 |
|  |  | Heat shock protein HSP 90 | HSP90AA1 | Q9NQ11 |
|  |  | Cell division protein kinase 2 | CDK2 | P25054 |
|  |  | Serine/threonine-protein kinase Chk1 | CHEK1 | P25054 |
|  |  | mRNA of PKA Catalytic Subunit C-alpha | PRKACA | P16615 |
|  |  | Proto-oncogene serine/threonine-protein kinase Pim-1 | PIM1 | P16615 |
|  |  | Cyclin-A2 | CCNA2 | P25054 |
|  |  | Calmodulin | CAMK1 | P25054 |
| Aloe-emodin | MOL000471 | Prostaglandin G/H synthase 1 | PTGS1 | P16615 |
|  |  | Prostaglandin G/H synthase 2 | PTGS2 | P16615 |
|  |  | Heat shock protein HSP 90 | HSP90AA1 | Q9NQ11 |
|  |  | Phosphatidylinositol-4,5-bisphosphate 3-kinase catalytic subunit, gamma isoform | PIK3CG | P16615 |
|  |  | mRNA of PKA Catalytic Subunit C-alpha | PRKACA | P16615 |
|  |  | Nuclear receptor coactivator 2 | NCOA2 | Q9NQ11 |
|  |  | cAMP-dependent protein kinase inhibitor alpha | PKIA | P16615 |
|  |  | Ig gamma-1 chain C region | IGHG1 | Q9NQ11 |
|  |  | Cyclin-dependent kinase inhibitor 1 | CDKN1B | P25054 |
|  |  | Eukaryotic translation initiation factor 6 | EIF6 | P50416 |
|  |  | Apoptosis regulator BAX | BAX | P25054 |
|  |  | Tumor necrosis factor | TNF | P61925 |
|  |  | Caspase-3 | CASP3 | P25054 |
|  |  | Cellular tumor antigen p53 | TP53 | P61925 |
|  |  | Fatty acid synthase | FASN | P20309 |
|  |  | Protein kinase C alpha type | PRKCA | P16615 |
|  |  | Protein kinase C epsilon type | PRKCE | P16615 |
|  |  | Proliferating cell nuclear antigen | PCNA | P16615 |
|  |  | Myc proto-oncogene protein | MYC | Q9NQ11 |
|  |  | Interleukin-1 beta | IL1B | Q9NQ11 |
|  |  | Protein kinase C delta type | PRKCD | P16615 |
|  |  | G2/mitotic-specific cyclin-B1 | CCNB1 | P25054 |
| Eugenol | MOL000254 | Prostaglandin G/H synthase 1 | PTGS1 | P16615 |
|  |  | Dopamine D1 receptor | DRD1 | P50416 |
|  |  | Muscarinic acetylcholine receptor M3 | CHRM3 | P25054 |
|  |  | Muscarinic acetylcholine receptor M1 | CHRM1 | P25054 |
|  |  | Beta-1 adrenergic receptor | ADRB1 | P08588 |
|  |  | Prostaglandin G/H synthase 2 | PTGS2 | P16615 |
|  |  | Nitric-oxide synthase, endothelial | NOS3 | Q9NQ11 |
|  |  | Alpha-2A adrenergic receptor | ADRA2A | P08913 |
|  |  | Alpha-2C adrenergic receptor | ADRA2C | D6RGL0 |
|  |  | Sodium-dependent noradrenaline transporter | SLC6A2 | P61925 |
|  |  | Alpha-1A adrenergic receptor | ADRA1A | B0ZBD3 |
|  |  | Muscarinic acetylcholine receptor M2 | CHRM2 | P25054 |
|  |  | Sodium-dependent dopamine transporter | SLC6A3 | P61925 |
|  |  | Beta-2 adrenergic receptor | ADRB2 | A2N4T7 |
|  |  | Beta-lactamase | BQ8482 | P25054 |
|  |  | Amine oxidase [flavin-containing] B | MAOB | Q9NQ11 |
|  |  | Amine oxidase [flavin-containing] A | MAOA | Q9NQ11 |
|  |  | Lysozyme | LYZD2 | Q9NQ11 |
|  |  | Chymotrypsinogen B | CTRB1 | P50416 |
|  |  | Alpha-1D adrenergic receptor | ADRA1D | B0ZBE0 |
|  |  | Leukotriene A-4 hydrolase | LTA4H | Q9NQ11 |
|  |  | Alpha-2B adrenergic receptor | ADRA2B | L8E8D5 |
|  |  | Urokinase-type plasminogen activator | PLAU | P16615 |
|  |  | Sodium channel protein type 5 subunit alpha | SCN5A | P16615 |
|  |  | Transcription factor p65 | RELA | P16615 |
|  |  | Cytochrome P450 1A1 | CYP1A1 | P50416 |
|  |  | Cytochrome P450 1B1 | CYP1B1 | P50416 |
|  |  | T-lymphocyte activation antigen CD86 | CD86 | P25054 |
|  |  | Serine/threonine-protein phosphatase 2B catalytic subunit alpha isoform | PPP3CA | P16615 |
|  |  | Mucin-1 | MUC1 | Q9NQ11 |
|  |  | Quinone oxidoreductase | CRYZ | P50416 |
|  |  | Calcium-transporting ATPase type 2C member 1 | ATP2C1 | P25054 |
|  |  | Short transient receptor potential channel 3 | TRPC3 | P61925 |
|  |  | Transient receptor potential cation channel subfamily V member 3 | TRPV3 | P61925 |
|  |  | Angiotensin-converting enzyme | ACE | P12821 |
|  |  | Ecto-NOX disulfide-thiol exchanger 2 | ENOX2 | P50416 |
| Emodin | MOL000472 | Prostaglandin G/H synthase 1 | PTGS1 | P16615 |
|  |  | Prostaglandin G/H synthase 2 | PTGS2 | P16615 |
|  |  | Coagulation factor VII | F7 | P20309 |
|  |  | Heat shock protein HSP 90 | HSP90AA1 | Q9NQ11 |
|  |  | Phosphatidylinositol-4,5-bisphosphate 3-kinase catalytic subunit, gamma isoform | PIK3CG | P16615 |
|  |  | mRNA of PKA Catalytic Subunit C-alpha | PRKACA | P16615 |
|  |  | Ig gamma-1 chain C region | IGHG1 | Q9NQ11 |
|  |  | Vascular endothelial growth factor receptor 2 | KDR | Q9NQ11 |
|  |  | DNA topoisomerase II | TOP2A | P61925 |
|  |  | Nuclear receptor coactivator 2 | NCOA2 | Q9NQ11 |
|  |  | Nuclear receptor coactivator 1 | NCOA1 | Q9NQ11 |
|  |  | Calmodulin | CALM1 | P25054 |
|  |  | Cyclin-dependent kinase inhibitor 1 | CDKN1B | P25054 |
|  |  | Vascular endothelial growth factor receptor 1 | FLT1 | P20309 |
|  |  | Matrix metalloproteinase-9 | MMP9 | Q9NQ11 |
|  |  | Pro-epidermal growth factor | EGF | P50416 |
|  |  | Tumor necrosis factor | TNF | P61925 |
|  |  | Caspase-3 | CASP3 | P25054 |
|  |  | Cellular tumor antigen p53 | TP53 | P61925 |
|  |  | Protein kinase C epsilon type | PRKCE | P16615 |
|  |  | Interstitial collagenase | MMP1 | Q9NQ11 |
|  |  | Peroxisome proliferator-activated receptor gamma | PPARG | P16615 |
|  |  | Myc proto-oncogene protein | MYC | Q9NQ11 |
|  |  | Cytochrome P450 1A1 | CYP1A1 | P50416 |
|  |  | Interleukin-1 beta | IL1B | Q9NQ11 |
|  |  | Protein kinase C delta type | PRKCD | P16615 |
|  |  | Granulocyte-macrophage colony-stimulating factor | CSF2 | P50416 |
|  |  | Transforming growth factor beta-1 | TGFB1 | P61925 |
|  |  | Actin, aortic smooth muscle | ACTA2 | D2JYH4 |
|  |  | Amine oxidase [flavin-containing] B | MAOB | Q9NQ11 |
|  |  | Tyrosine-protein kinase BTK | BTK | P25054 |
|  |  | Solute carrier family 2, facilitated glucose transporter member 4 | SLC2A4 | P61925 |
|  |  | Vascular endothelial growth factor receptor 3 | FLT4 | P20309 |
|  |  | Solute carrier family 2, facilitated glucose transporter member 1 | SLC2A1 | P61925 |
| Spathulenol | MOL004706 | Muscarinic acetylcholine receptor M3 | CHRM3 | P25054 |
|  |  | Muscarinic acetylcholine receptor M1 | CHRM1 | P25054 |
|  |  | Muscarinic acetylcholine receptor M2 | CHRM2 | P25054 |
|  |  | Gamma-aminobutyric acid receptor subunit alpha-1 | GABRA1 | P20309 |
| Kaempferol | MOL000422 | Nitric oxide synthase, inducible | NOS2 | Q9NQ11 |
|  |  | Prostaglandin G/H synthase 1 | PTGS1 | P16615 |
|  |  | Peroxisome proliferator activated receptor gamma | PPARG | P16615 |
|  |  | Prostaglandin G/H synthase 2 | PTGS2 | P16615 |
|  |  | Heat shock protein HSP 90 | HSP90AA1 | Q9NQ11 |
|  |  | Phosphatidylinositol-4,5-bisphosphate 3-kinase catalytic subunit, gamma isoform | PIK3CG | P16615 |
|  |  | mRNA of PKA Catalytic Subunit C-alpha | PRKACA | P16615 |
|  |  | Nuclear receptor coactivator 2 | NCOA2 | Q9NQ11 |
|  |  | Dipeptidyl peptidase IV | DPP4 | P50416 |
|  |  | Trypsin-1 | PRSS1 | P16615 |
|  |  | Progesterone receptor | PGR | P16615 |
|  |  | Muscarinic acetylcholine receptor M1 | CHRM1 | P25054 |
|  |  | Nitric-oxide synthase, endothelial | NOS3 | Q9NQ11 |
|  |  | Gamma-aminobutyric-acid receptor alpha-2 subunit | GABRA2 | P20309 |
|  |  | Sodium-dependent noradrenaline transporter | SLC6A2 | P61925 |
|  |  | Muscarinic acetylcholine receptor M2 | CHRM2 | P25054 |
|  |  | Trypsin-1 | PRSS1 | P16615 |
|  |  | Progesterone receptor | PGR | P16615 |
|  |  | Muscarinic acetylcholine receptor M1 | CHRM1 | P25054 |
|  |  | Nitric-oxide synthase, endothelial | NOS3 | Q9NQ11 |
|  |  | Gamma-aminobutyric-acid receptor alpha-2 subunit | GABRA2 | P20309 |
|  |  | Sodium-dependent noradrenaline transporter | SLC6A2 | P61925 |
|  |  | Muscarinic acetylcholine receptor M2 | CHRM2 | P25054 |
|  |  | Transcription factor AP-1 | JUN | Q9NQ11 |
|  |  | Activator of 90 kDa heat shock protein ATPase homolog 1 | AHSA1 | G3V3W9 |
|  |  | Caspase-3 | CASP3 | P25054 |
|  |  | Mitogen-activated protein kinase 8 | MAPK8 | Q9NQ11 |
|  |  | Xanthine dehydrogenase/oxidase | XDH | P61925 |
|  |  | Signal transducer and activator of transcription 1-alpha/beta | STAT1 | P61925 |
|  |  | Peroxisome proliferator-activated receptor gamma | PPARG | P16615 |
|  |  | Heme oxygenase 1 | HMOX1 | Q9NQ11 |
|  |  | Cytochrome P450 3A4 | CYP3A4 | P50416 |
|  |  | Cytochrome P450 1A2 | CYP1A2 | P50416 |
|  |  | Cytochrome P450 1A1 | CYP1A1 | P50416 |
|  |  | Intercellular adhesion molecule 1 | ICAM1 | Q9NQ11 |
|  |  | E-selectin | SELE | P16615 |
|  |  | Vascular cell adhesion protein 1 | VCAM1 | P61925 |
|  |  | Nuclear receptor subfamily 1 group I member 2 | NR1I2 | P16615 |
|  |  | Cytochrome P450 1B1 | CYP1B1 | P50416 |
|  |  | Hyaluronan synthase 2 | HAS2 | Q9NQ11 |
|  |  | Glutathione S-transferase P | GSTP1 | Q9NQ11 |
|  |  | 26S proteasome non-ATPase regulatory subunit 3 | PSMD3 | P16615 |
|  |  | Solute carrier family 2, facilitated glucose transporter member 4 | SLC2A4 | P61925 |
|  |  | Nuclear receptor subfamily 1 group I member 3 | NR1I3 | P16615 |
|  |  | Insulin receptor | INSR | Q9NQ11 |
|  |  | Type I iodothyronine deiodinase | DIO1 | P50416 |
|  |  | Serine/threonine-protein phosphatase 2B catalytic subunit alpha isoform | PPP3CA | P16615 |
|  |  | Glutathione S-transferase Mu 1 | GSTM1 | Q9NQ11 |
|  |  | Glutathione S-transferase Mu 2 | GSTM2 | Q9NQ11 |
| Isorhamnetin | MOL000354 | Nitric oxide synthase, inducible | NOS2 | Q9NQ11 |
|  |  | Prostaglandin G/H synthase 1 | PTGS1 | P16615 |
|  |  | Estrogen receptor | ESR | P20309 |
|  |  | Peroxisome proliferator activated receptor gamma | PPARG | P16615 |
|  |  | Prostaglandin G/H synthase 2 | PTGS2 | P16615 |
|  |  | mRNA of Protein-tyrosine phosphatase, non-receptor type 1 | PTPN1 | P16615 |
|  |  | Estrogen receptor beta | ESR2 | P20309 |
|  |  | Dipeptidyl peptidase IV | DPP4 | P50416 |
|  |  | Mitogen-activated protein kinase 14 | MAPK14 | Q9NQ11 |
|  |  | Glycogen synthase kinase-3 beta | GSK3B | P49841 |
|  |  | Heat shock protein HSP 90 | HSP90AA1 | Q9NQ11 |
|  |  | Cell division protein kinase 2 | CDK2 | P25054 |
|  |  | Phosphatidylinositol-4,5-bisphosphate 3-kinase catalytic subunit, gamma isoform | PIK3CG | P16615 |
|  |  | mRNA of PKA Catalytic Subunit C-alpha | PRKACA | P16615 |
|  |  | Trypsin-1 | PRSS1 | P16615 |
|  |  | Proto-oncogene serine/threonine-protein kinase Pim-1 | PIM1 | P16615 |
|  |  | Cyclin-A2 | CCNA2 | P25054 |
|  |  | Nuclear receptor coactivator 2 | NCOA2 | Q9NQ11 |
|  |  | Calmodulin | CALM1 | P25054 |
|  |  | Glycogen phosphorylase, muscle form | PYGM | P16615 |
|  |  | Peroxisome proliferator activated receptor delta | PPARD | P16615 |
|  |  | Serine/threonine-protein kinase Chk1 | CHEK1 | P25054 |
|  |  | Nuclear receptor coactivator 1 | NCOA1 | Q9NQ11 |
|  |  | Coagulation factor VII | F7 | P20309 |
|  |  | Nitric-oxide synthase, endothelial | NOS3 | Q9NQ11 |
|  |  | Gamma-aminobutyric acid receptor subunit alpha-1 | GABRA1 | P20309 |
|  |  | Amine oxidase [flavin-containing] B | MAOB | Q9NQ11 |
|  |  | Glutamate receptor 2 | GRIA2 | P20309 |
|  |  | Transcription factor p65 | RELA | P16615 |
|  |  | Xanthine dehydrogenase/oxidase | XDH | P61925 |
|  |  | Neutrophil cytosol factor 1 | NCF1 | Q9NQ11 |
|  |  | Oxidized low-density lipoprotein receptor 1 | OLR1 | P16615 |
| isoliquiritigenin | MOL001789 | Prostaglandin G/H synthase 1 | PTGS1 | P16615 |
|  |  | Estrogen receptor | ESR1 | P20309 |
|  |  | Peroxisome proliferator activated receptor gamma | PPARG | P16615 |
|  |  | Prostaglandin G/H synthase 2 | PTGS2 | P16615 |
|  |  | Carbonic anhydrase II | CA2 | P25054 |
|  |  | Beta-2 adrenergic receptor | ADRB2 | A2N4T7 |
|  |  | Estrogen receptor beta | ESR2 | P20309 |
|  |  | Mitogen-activated protein kinase 14 | MAPK14 | Q9NQ11 |
|  |  | Glycogen synthase kinase-3 beta | GSK3B | P49841 |
|  |  | Heat shock protein HSP 90 | HSP90AA1 | Q9NQ11 |
|  |  | Cell division protein kinase 2 | CDK2 | P25054 |
|  |  | Amine oxidase [flavin-containing] B | MAOB | Q9NQ11 |
|  |  | mRNA of PKA Catalytic Subunit C-alpha | PRKACA | P16615 |
|  |  | cAMP-dependent protein kinase inhibitor alpha | PKIA | P16615 |
|  |  | Nitric oxide synthase, inducible | NOS2 | Q9NQ11 |
|  |  | Phosphatidylinositol-4,5-bisphosphate 3-kinase catalytic subunit, gamma isoform | PIK3CG | P16615 |
|  |  | Beta-lactamase | BQ8482 | P25054 |
|  |  | Cyclin-A2 | CCNA2 | P25054 |
|  |  | Nuclear receptor coactivator 2 | NCOA2 | Q9NQ11 |
|  |  | Proto-oncogene c-Fos | FOS | P20309 |
|  |  | Apoptosis regulator BAX | BAX | P25054 |
|  |  | E-selectin | SELE | P16615 |
|  |  | Vascular cell adhesion protein 1 | VCAM1 | P61925 |
|  |  | Gamma-aminobutyric acid type B receptor subunit 1 | GABBR1 | P20309 |
|  |  | Tyrosinase | TYR | P61925 |
|  |  | Junctional adhesion molecule A | F11R | P20309 |
|  |  | Tyrosine-protein kinase JAK2 | JAK2 | Q9NQ11 |
|  |  | Metallothionein-2 | MT2A | Q9NQ11 |
|  |  | Solute carrier family 2, facilitated glucose transporter member 1 | SLC2A1 | P61925 |
| Angelicin | MOL003590 | Cytochrome P450 1A1 | CYP1A1 | P50416 |
| Cerevisterol | MOL000279 | Mineralocorticoid receptor | NR3C2 | P16615 |
| β-amyrin acetate | MOL000286 | Prostaglandin G/H synthase 2 | PTGS2 | P16615 |
|  |  | Prostaglandin G/H synthase 1 | PTGS1 | P16615 |
|  |  | Amine oxidase [flavin-containing] B | MAOB | Q9NQ11 |
|  |  | Amine oxidase [flavin-containing] A | MAOA | Q9NQ11 |
| (-)-Hesperetin | MOL002341 | Prostaglandin G/H synthase 1 | PTGS1 | P16615 |
|  |  | Sodium channel protein type 5 subunit alpha | SCN5A | P16615 |
|  |  | Prostaglandin G/H synthase 2 | PTGS2 | P16615 |
|  |  | Heat shock protein HSP 90 | HSP90AA1 | Q9NQ11 |
|  |  | Phosphatidylinositol-4,5-bisphosphate 3-kinase catalytic subunit, gamma isoform | PIK3CG | P16615 |
|  |  | mRNA of PKA Catalytic Subunit C-alpha | PRKACA | P16615 |
|  |  | Nuclear receptor coactivator 2 | NCOA2 | Q9NQ11 |
|  |  | Nuclear receptor coactivator 1 | NCOA1 | Q9NQ11 |
|  |  | Calmodulin | CALM1 | P25054 |
| Rosmarinic acid | MOL011865 | Estrogen receptor | ESR1 | P20309 |
|  |  | Peroxisome proliferator activated receptor gamma | PPARG | P16615 |
|  |  | Prostaglandin G/H synthase 2 | PTGS2 | P16615 |
|  |  | Dipeptidyl peptidase IV | DPP4 | P50416 |
|  |  | Trypsin-1 | PRSS1 | P16615 |
|  |  | Transcription factor p65 | RELA | P16615 |
|  |  | Inhibitor of nuclear factor kappa-B kinase subunit beta | IKBKB | Q9NQ11 |
|  |  | Cyclin-dependent kinase inhibitor 1 | CDKN1B | P25054 |
|  |  | Eukaryotic translation initiation factor 6 | EIF6 | P50416 |
|  |  | Mitogen-activated protein kinase 1 | MAP3K1 | Q9NQ11 |
|  |  | Caspase-3 | CASP3 | P25054 |
|  |  | Signal transducer and activator of transcription 1-alpha/beta | STAT1 | P61925 |
|  |  | C-C motif chemokine 2 | CCL2 | P25054 |
|  |  | Maltase-glucoamylase, intestinal | MGAM | Q9NQ11 |
|  |  | Interleukin-2 | IL2 | Q9NQ11 |
|  |  | Nuclear factor of activated T-cells, cytoplasmic 3 | NFATC3 | Q9NQ11 |
|  |  | G1/S-specific cyclin-D3 | CCND3 | P25054 |
|  |  | Interleukin-4 | IL4 | Q9NQ11 |
|  |  | Interleukin-5 | IL5 | Q9NQ11 |
|  |  | C-C motif chemokine 3 | CCL3 | P25054 |
|  |  | T-lymphocyte activation antigen CD80 | CD80 | P25054 |
|  |  | T-lymphocyte activation antigen CD86 | CD86 | P25054 |
|  |  | Eotaxin | Eotaxin | P50416 |
|  |  | C-C chemokine receptor type 3 | CCR3 | P25054 |
|  |  | Indoleamine 2,3-dioxygenase 1 | IDO1 | Q9NQ11 |
|  |  | Alpha-synuclein | SNCA | P61925 |
|  |  | Ig gamma-1 chain C region | IGHG1 | Q9NQ11 |
|  |  | T-cell surface glycoprotein CD3 zeta chain | CD247 | P25054 |
|  |  | Complement C3 | C3 | P25054 |
|  |  | Prothrombin | F2 | P20309 |
|  |  | Polyunsaturated fatty acid 5-lipoxygenase | ALOX5 | [P09917](https://www.uniprot.org/uniprot/P09917) |
|  |  | 5-hydroxytryptamine receptor 1A | HTR1A | [P08908](https://www.uniprot.org/uniprot/P08908) |
|  |  | Complement C5 | C5 | P25054 |
| Aesculetin | MOL003837 | Apoptosis regulator Bcl-2 | BCL2 | P25054 |
|  |  | Cyclin-dependent kinase inhibitor 1 | CDKN1B | P25054 |
|  |  | Retinoblastoma-associated protein | RB1 | P16615 |
|  |  | Caspase-3 | CASP3 | P25054 |
|  |  | Interstitial collagenase | MMP1 | Q9NQ11 |
|  |  | Stromelysin-1 | MMP3 | Q9NQ11 |
|  |  | Transcription factor E2F1 | E2F1 | P50416 |
| Diosmin | MOL005093 | Prostaglandin G/H synthase 1 | PTGS1 | P16615 |
|  |  | Prostaglandin G/H synthase 2 | PTGS2 | P16615 |
|  |  | mRNA of Protein-tyrosine phosphatase, non-receptor type 1 | PTPN1 | P16615 |
|  |  | Dipeptidyl peptidase IV | DPP4 | P50416 |
|  |  | Heat shock protein HSP 90 | HSP90AA1 | Q9NQ11 |
|  |  | mRNA of PKA Catalytic Subunit C-alpha | PRKACA | P16615 |
|  |  | Trypsin-1 | PRSS1 | P16615 |
|  |  | Nuclear receptor coactivator 2 | NCOA2 | Q9NQ11 |
|  |  | Nuclear receptor coactivator 1 | NCOA1 | Q9NQ11 |
|  |  | Calmodulin | CALM1 | P25054 |
| Atractylenolide-1 | MOL000043 | Gamma-aminobutyric-acid receptor alpha-2 subunit | GABRA2 | P20309 |
|  |  | Gamma-aminobutyric acid receptor subunit alpha-1 | GABRA1 | P20309 |
|  |  | Neuronal acetylcholine receptor protein, alpha-7 chain | CHRNA2 | P25054 |
|  |  | Vascular endothelial growth factor A | VEGFA | P61925 |
|  |  | Tumor necrosis factor | TNF | P61925 |
|  |  | Interleukin-6 | IL6 | Q9NQ11 |
|  |  | Interleukin-1 beta | IL1B | Q9NQ11 |
|  |  | Placenta growth factor | PGF | P16615 |
| Saikosaponin A | MOL004635 | Apoptosis regulator Bcl-2 | BCL2 | P25054 |
|  |  | Apoptosis regulator BAX | BAX | P25054 |
|  |  | Retinoblastoma-associated protein | RB1 | P16615 |
|  |  | Caspase-3 | CASP3 | P25054 |
|  |  | Myc proto-oncogene protein | MYC | Q9NQ11 |
| 4-Hydroxy-3-butylphthalide | MOL002181 | Prostaglandin G/H synthase 1 | PTGS1 | P16615 |
|  |  | Muscarinic acetylcholine receptor M3 | CHRM3 | P25054 |
|  |  | Muscarinic acetylcholine receptor M1 | CHRM1 | P25054 |
|  |  | Beta-1 adrenergic receptor | ADRB1 | P08588 |
|  |  | Sodium channel protein type 5 subunit alpha | SCN5A | P16615 |
|  |  | Prostaglandin G/H synthase 2 | PTGS2 | P16615 |
|  |  | Alpha-2A adrenergic receptor | ADRA2A | P08913 |
|  |  | Alpha-2C adrenergic receptor | ADRA2C | D6RGL0 |
|  |  | CGMP-inhibited 3',5'-cyclic phosphodiesterase A | PDE3A | P16615 |
|  |  | 5-hydroxytryptamine 2A receptor | HTR2A | Q9NQ11 |
|  |  | Sodium-dependent noradrenaline transporter | SLC6A2 | P61925 |
|  |  | Alpha-1A adrenergic receptor | ADRA1A | B0ZBD3 |
|  |  | Muscarinic acetylcholine receptor M2 | CHRM2 | P25054 |
|  |  | Alpha-2B adrenergic receptor | ADRA2B | L8E8D5 |
|  |  | Sodium-dependent dopamine transporter | SLC6A3 | P61925 |
|  |  | Sodium-dependent serotonin transporter | SLC6A4 | P61925 |
|  |  | D(2) dopamine receptor | DRD2 | P50416 |
|  |  | Gamma-aminobutyric acid receptor subunit alpha-1 | GABRA1 | P20309 |
|  |  | Heat shock protein HSP 90 | HSP90AA1 | Q9NQ11 |
|  |  | Amine oxidase [flavin-containing] B | MAOB | Q9NQ11 |
|  |  | mRNA of PKA Catalytic Subunit C-alpha | PRKACA | P16615 |
|  |  | cAMP-dependent protein kinase inhibitor alpha | PKIA | P16615 |
| Tangeretin (6CI) | MOL005814 | Nitric oxide synthase, inducible | NOS2 | Q9NQ11 |
|  |  | Prostaglandin G/H synthase 1 | PTGS1 | P16615 |
|  |  | Potassium voltage-gated channel subfamily H member 2 | KCNH2 | Q9NQ11 |
|  |  | Sodium channel protein type 5 subunit alpha | SCN5A | P16615 |
|  |  | Prostaglandin G/H synthase 2 | PTGS2 | P16615 |
|  |  | Coagulation factor VII | F7 | P20309 |
|  |  | Beta-2 adrenergic receptor | ADRB2 | A2N4T7 |
|  |  | DNA topoisomerase II | TOP2A | P61925 |
|  |  | Estrogen receptor beta | ESR2 | P20309 |
|  |  | Dipeptidyl peptidase IV | DPP4 | P50416 |
|  |  | Heat shock protein HSP 90 | HSP90AA1 | Q9NQ11 |
|  |  | Serine/threonine-protein kinase Chk1 | CHEK1 | P25054 |
|  |  | Trypsin-1 | PRSS1 | P16615 |
|  |  | Nuclear receptor coactivator 2 | NCOA2 | Q9NQ11 |
|  |  | Calcium-activated potassium channel subunit alpha 1 | KCNMA1 | Q9NQ11 |
|  |  | Calmodulin | CAMK1 | P25054 |
|  |  | Nitric-oxide synthase, endothelial | NOS3 | Q9NQ11 |
|  |  | Cyclin-dependent kinase inhibitor 1 | CDKN1B | P25054 |
|  |  | Eukaryotic translation initiation factor 6 | EIF6 | P50416 |
|  |  | Cell division protein kinase 2 | CDK2 | P25054 |
|  |  | Cell division protein kinase 4 | CDK4 | P25054 |
|  |  | 78 kDa glucose-regulated protein | HSPA5 | Q9NQ11 |
|  |  | Heme oxygenase 1 | HMOX1 | Q9NQ11 |
|  |  | Cytochrome P450 1A1 | CYP1A1 | P50416 |
| Menthyl benzoate | MOL006219 | Lysozyme | LYZD2 | Q9NQ11 |
| β-Cyperol | MOL004036 | Gamma-aminobutyric-acid receptor alpha-2 subunit | GABRA2 | P20309 |
| Tangeretin | MOL005814 | Nitric oxide synthase, inducible | NOS2 | Q9NQ11 |
|  |  | Prostaglandin G/H synthase 1 | PTGS1 | P16615 |
|  |  | Potassium voltage-gated channel subfamily H member 2 | KCNH2 | Q9NQ11 |
|  |  | Sodium channel protein type 5 subunit alpha | SCN5A | P16615 |
|  |  | Prostaglandin G/H synthase 2 | PTGS2 | P16615 |
|  |  | Coagulation factor VII | F7 | P20309 |
|  |  | Beta-2 adrenergic receptor | ADRB2 | A2N4T7 |
|  |  | DNA topoisomerase II | TOP2A | P61925 |
|  |  | Estrogen receptor beta | ESR2 | P20309 |
|  |  | Dipeptidyl peptidase IV | DPP4 | P50416 |
|  |  | Heat shock protein HSP 90 | HSP90AA1 | Q9NQ11 |
|  |  | Serine/threonine-protein kinase Chk1 | CHEK1 | P25054 |
|  |  | Trypsin-1 | PRSS1 | P16615 |
|  |  | Nuclear receptor coactivator 2 | NCOA2 | Q9NQ11 |
|  |  | Calcium-activated potassium channel subunit alpha 1 | KCNMA1 | Q9NQ11 |
|  |  | Calmodulin | CAMK1 | P25054 |
|  |  | Nitric-oxide synthase, endothelial | NOS3 | Q9NQ11 |
|  |  | Cyclin-dependent kinase inhibitor 1 | CDKN1B | P25054 |
|  |  | Eukaryotic translation initiation factor 6 | EIF6 | P50416 |
|  |  | Cell division protein kinase 2 | CDK2 | P25054 |
|  |  | Cell division protein kinase 4 | CDK4 | P25054 |
|  |  | 78 kDa glucose-regulated protein | HSPA5 | Q9NQ11 |
|  |  | Heme oxygenase 1 | HMOX1 | Q9NQ11 |
|  |  | Brain-derived neurotrophic factor | BDNF | [P23560](https://www.uniprot.org/uniprot/P23560) |
|  |  | Angiotensin-converting enzyme | ACE | P12821 |
|  |  | 5-hydroxytryptamine receptor 1A | HTR1A | [P08908](https://www.uniprot.org/uniprot/P08908) |
|  |  | Cytochrome P450 1A1 | CYP1A1 | P50416 |
| 5,6,7,8-Tetramethoxy-2-(4-methoxyphenyl)-4-benzopyrone | MOL005814 | Nitric oxide synthase, inducible | NOS2 | Q9NQ11 |
|  |  | Prostaglandin G/H synthase 1 | PTGS1 | P16615 |
|  |  | Potassium voltage-gated channel subfamily H member 2 | KCNH2 | Q9NQ11 |
|  |  | Sodium channel protein type 5 subunit alpha | SCN5A | P16615 |
|  |  | Prostaglandin G/H synthase 2 | PTGS2 | P16615 |
|  |  | Coagulation factor VII | F7 | P20309 |
|  |  | Beta-2 adrenergic receptor | ADRB2 | A2N4T7 |
|  |  | DNA topoisomerase II | TOP2A | P61925 |
|  |  | Estrogen receptor beta | ESR2 | P20309 |
|  |  | Dipeptidyl peptidase IV | DPP4 | P50416 |
|  |  | Heat shock protein HSP 90 | HSP90AA1 | Q9NQ11 |
|  |  | Serine/threonine-protein kinase Chk1 | CHEK1 | P25054 |
|  |  | Trypsin-1 | PRSS1 | P16615 |
|  |  | Nuclear receptor coactivator 2 | NCOA2 | Q9NQ11 |
|  |  | Calcium-activated potassium channel subunit alpha 1 | KCNMA1 | Q9NQ11 |
|  |  | Calmodulin | CAMK1 | P25054 |
|  |  | Nitric-oxide synthase, endothelial | NOS3 | Q9NQ11 |
|  |  | Cyclin-dependent kinase inhibitor 1 | CDKN1B | P25054 |
|  |  | Eukaryotic translation initiation factor 6 | EIF6 | P50416 |
|  |  | Cell division protein kinase 2 | CDK2 | P25054 |
|  |  | Cell division protein kinase 4 | CDK4 | P25054 |
|  |  | 78 kDa glucose-regulated protein | HSPA5 | Q9NQ11 |
|  |  | Heme oxygenase 1 | HMOX1 | Q9NQ11 |
|  |  | Cytochrome P450 1A1 | CYP1A1 | P50416 |
