# Supplementary material for: An Integrative Pharmacology-Based Strategy to Uncover the Mechanism of Xiong-Pi-Fang in Treating Coronary Heart Disease with Depression
Source: Front Pharmacol. 2021 Apr 1;12:590602. doi: 10.3389/fphar.2021.590602 (PMC8048422; doi:10.3389/fphar.2021.590602)
Supplement: Supplementary file 5 [file Image1.jpeg]

Frontiers | An Integrative Pharmacology-Based Strategy to Uncover the Mechanism of Xiong-Pi-Fang Against Coronary Heart Disease with Depression | Pharmacology


- About
- Journals
- Research Topics
- Articles
- More

Submit

My Frontiers

Office

- TSOF
  - TSOF
  - Article Production

Typesetter 3

frontiersproduction@tnq.co.in

- Profile
- Settings & Privacy
- Help Center
- Logout

Submit

**Impact Factor 4.225** | **CiteScore 5.0**More on impact ›

|  |  |
| --- | --- |
| Frontiers in Pharmacology | Ethnopharmacology |

Toggle navigation


Section


- (current)Section
- About
- Articles
- Research topics
- For authors 
  - Why submit?
  - Fees
  - Article types
  - Author guidelines
  - Review guidelines
  - Submission checklist
  - Contact editorial office
  - Submit your manuscript
- Editorial board

- *Article alerts*

##### This article is part of the Research Topic

Integrative Pharmacology-based Research on Traditional Medicine: Methodologies, Medical and Pharmacological Applications
View all
49
Articles

Articles


**Suggest a Research Topic >**

- 97
  total views

 View Article Impact

**Suggest a Research Topic >**

##### SHARE ON

- Facebook

  0
- Twitter

  0
- LinkedIn

  0
- AddThis

  New


## Original Research ARTICLE

Front. Pharmacol.
| doi: 10.3389/fphar.2021.590602

# An Integrative Pharmacology-Based Strategy to Uncover the Mechanism of Xiong-Pi-Fang Against Coronary Heart Disease with Depression Provisionally accepted The final, formatted version of the article will be published soon. **Notify me**

Lihong Zhang1\*,  Yu Zhang1,  Mingdan Zhu2, Limin Pei1, 
Fangjun Deng1, 
 Jinhong Chen1, Shaoqiang Zhang2, 
Zidong Cong2, 
Wuxun Du2\* and 
Xuefeng Xiao1\*

- 1Tianjin University of Traditional Chinese Medicine, China
- 2Second Affiliated Hospital of Tianjin University of Traditional Chinese Medicine, China

Objectives: This study aimed to explore the mechanism of Xiong-Pi-Fang (XPF) in the treatment of coronary heart disease (CHD) with depression by an integrative strategy combining serum pharmacochemistry, network pharmacology analysis, and experimental validation.A UPLC-Q-TOF/MS method was constructed to identify compounds in rat serum after oral administration of XPF, and a component-target network was established using Cytoscape, between the targets of XPF ingredients and CHD with depression. Furthermore, Gene Ontology and Kyoto Encyclopedia of Genes and Genomes pathway enrichment analyses were performed to deduce the mechanism of XPF against CHD with depression. Finally, in a chronic unpredictable mild stress (CUMS)-and isoproterenol (ISO)-induced rat model, TUNEL was used to detect the apoptosis index of the myocardium and hippocampus, and ELISA and western blot were used to detect the predicted hub targets, namely AngⅡ, 5-HT, cAMP, PKA, CREB, BDNF, Bcl-2, Bax, Cyt-c, and caspase-3.We identified 51 compounds in rat serum after oral administration of XPF, which mainly included phenolic acids, saponins, and flavonoids. Network pharmacology analysis revealed that XPF may regulate targets, such as ACE2, HTR1A, HTR2A, AKT1, PKIA, CREB1, BDNF, BCL2, BAX, CASP3, cAMP signaling pathway, and cell apoptosis process in the treatment of CHD with depression. ELISA analysis showed that XPF decreased Ang-II content in the circulation and central system, inhibited 5-HT levels in peripheral circulation, and increased 5-HT content in the central nervous system and cAMP content in the myocardia and hippocampus. Meanwhile, western blot analysis indicated that XPF could upregulate the expression levels of PKA, CREB, and BDNF both in the myocardia and hippocampus. TUNEL staining indicated that the apoptosis index of myocardial and hippocampal cells increased in CUMS-and ISO-induced CHD in rats under depression, and XPF could increase the expression of Bcl-2, inhibit the expression of Bax, Cyt-c, and caspase-3, and rectify the injury of the hippocampus and myocardium, which exerted antidepressant and antimyocardial ischemia effects.

Keywords: 
Integrative Pharmacology, Serum pharmacochemistry, network pharmacology analysis, Xiong-Pi-Fang, coronary heart disease with depression

Received: 02 Aug 2020;
Accepted: 11 Feb 2021.

Copyright: © 2021 Zhang, Zhang, Zhu, Pei, Deng, Chen, Zhang, Cong, Du and Xiao. This is an open-access article distributed under the terms of the Creative Commons Attribution License (CC BY). The use, distribution or reproduction in other forums is permitted, provided the original author(s) and the copyright owner(s) are credited and that the original publication in this journal is cited, in accordance with accepted academic practice. No use, distribution or reproduction is permitted which does not comply with these terms.

\* Correspondence: 
  
 Dr. Lihong Zhang, Tianjin University of Traditional Chinese Medicine, Tianjin, China, 1174048211@qq.com   
 Prof. Wuxun Du, Second Affiliated Hospital of Tianjin University of Traditional Chinese Medicine, Tianjin, Hebei, China, cnduwux@163.com   
 Prof. Xuefeng Xiao, Tianjin University of Traditional Chinese Medicine, Tianjin, China, kai1219@163.com

Write a comment...

Add

##### COMMENTARY

##### ORIGINAL ARTICLE

##### People also looked at

## Integrating Pharmacokinetics Study, Network Analysis, and Experimental Validation to Uncover the Mechanism of Qiliqiangxin Capsule Against Chronic Heart Failure

Yu Zhang, Mingdan Zhu, Fugeng Zhang, Shaoqiang Zhang, Wuxun Du and Xuefeng Xiao

## Galectin-3 Mediated Inflammatory Response Contributes to Neurological Recovery by QiShenYiQi in Subacute Stroke Model

Yule Wang, Shuang He, xin yan Liu, Li Zhi Xiong, Lin Zhu, Guangxu Xiao, DU XIAO LI, Hongxia Du, Wen Zhang, Yiqian Zhang, John Owoicho ORGAH, Yuxin Feng, Boli Zhang and Yan Zhu

## An Integrative Pharmacology-Based Approach for Evaluating the Potential Effects of Purslane Seed in Diabetes Mellitus Treatment Using UHPLC-LTQ-Orbitrap and TCMIP V2.0

Jinli Hou, Xiang Zhou, Ping Wang, Chunhui Zhao, Yuewen Qin, Feng Liu, Liping Yu and Haiyu Xu

## An Integrative Pharmacology-Based Pattern to Uncover the Pharmacological Mechanism of Ginsenoside H Dripping Pills in the Treatment of Depression

Libin Zhao, Rui Guo, Ningning Cao, Yingxian Lin, Wenjing Yang, Shuai Pei, Xiaowei Ma, Yu Zhang, Yingpeng Li, Zhaohui Song, Wuxun Du, Xuefeng Xiao and Changxiao Liu

## Integrating Pharmacology and Gut Microbiota Analysis to Explore the Mechanism of Citri Reticulatae Pericarpium Against Reserpine-Induced Spleen Deficiency in Rats

Yuying Zheng, Xuan Zeng, Pan Chen, Tingting Chen, Wei Peng and Weiwei Su

**Suggest a Research Topic >**

×

#### Supplementary Material

  

There is no supplementary material currently available for this article

Loading supplemental data...

  

|  | File Name |  |
| --- | --- | --- |
|  | Table 1.DOCX |  |
|  | Table 2.DOCX |  |
|  | Table 3.DOCX |  |
|  | Table 4.DOCX |  |
|  | Image 1.JPEG |  |

  

Close

- About Frontiers
- Institutional Membership
- Books
- News
- Frontiers' social media
- Contact
- Careers
- Submit
- Newsletter
- Help Center
- Terms & Conditions
- Privacy Policy

© 2007 - 2021 Frontiers Media S.A. All Rights Reserved

### Privacy Preference Center

Our website uses cookies that are necessary for its operation. Additional cookies are only used with your consent. These cookies are used to store and access information such as the characteristics of your device as well as certain personal data (IP address, navigation usage, geolocation data) and we process them to analyse the traffic on our website in order to provide you a better user experience, evaluate the efficiency of our communications and to personalise content to your interests. Some cookies are placed by third-party companies with which we work to deliver relevant ads on social media and the internet. Click on the different categories' headings to change your cookie preferences. Click on "More Information" if you wish to learn more about how data is collected and shared.
More information

### Manage Consent Preferences

#### Strictly Necessary Cookies

Always Active

These cookies are necessary for the website to function and cannot be switched off in our systems. They are usually only set in response to actions made by you which amount to a request for services, such as setting your privacy preferences, logging in or filling in forms. You can set your browser to block or alert you about these cookies, but some parts of the site will not then work. These cookies do not store any personally identifiable information.

#### Analytics Cookies

Analytics Cookies

These cookies allow us to count visits and traffic sources so we can measure and improve the performance of our site. They help us analyse which pages are the most and least popular and see how visitors move around the site.    All information these cookies collect is aggregated and therefore anonymous.

#### Functional Cookies

Functional Cookies

These cookies enable the website to provide enhanced functionality and personalisation. They may be set by us or by third party providers whose services we have added to our pages. If you do not allow these cookies then some or all of these services may not function properly.

#### Advertising Cookies

Advertising Cookies

These cookies may be set through our site by our advertising partners. They may be used by those companies to build a profile of your interests and show you relevant adverts on other sites.    They do not store directly personal information, but are based on uniquely identifying your browser and internet device. If you do not allow these cookies, you will experience less targeted advertising.

### Back Button Performance Cookies

Vendor Search  Search Icon

Filter Icon

Clear

checkbox label label

Apply Cancel

Consent Leg.Interest

checkbox label label

checkbox label label

checkbox label label

Confirm My Choices
